# Synthesis and characterization of novel nitrofurazanyl ethers as potential energetic plasticizers†

**DOI:** 10.1039/d5ra01282a

**Published:** 2025-04-22

**Authors:** Patrick Lieber, Uwe Schaller, Thomas M. Klapötke

**Affiliations:** a Fraunhofer Institute for Chemical Technology Joseph-von-Fraunhofer-Str. 7 76327 Pfinztal Germany uwe.schaller@ict.fraunhofer.de; b Department of Chemistry, Ludwig-Maximilian-University of Munich Butenandtstr. 5-13 81377 Munich Germany tmk@cup.uni-muenchen.de

## Abstract

Energetic plasticizers are used to improve the mechanical properties of advanced energetic formulations while increasing the overall energy content. Although nitro-1,2,5-oxadiazoles (nitrofurazans) possess excellent energetic properties such as a favorable oxygen balance and high heat of formation, their use as plasticizers has received little attention in the scientific literature. Four nitrofurazanyl ethers were synthesized by substitution of dinitrofurazan with linear alkoxides. The synthesized compounds were extensively analyzed by Fourier-transform infrared (FT-IR) spectroscopy, Raman spectroscopy, differential scanning calorimetry (DSC), thermogravimetric analysis (TGA), electrospray ionization (ESI) mass spectroscopy, mechanical sensitivity test, ^1^H nuclear magnetic resonance (NMR) spectroscopy and ^13^C NMR spectroscopy. They have lower mechanical sensitivity (>40 J) compared to modern energetic plasticizers in use, including 2,2-dinitropropyl formal/acetal (BDNPA/F), *n*-butylnitratoethylnitramine (BuNENA), and dinitrodiazaalkane (DNDA-57). In addition, the most promising compound 3-(2-(2-(2-azidoethoxy)ethoxy)ethoxy)-4-nitro-1,2,5-oxadiazole (NFPEG3N3) exhibits competitive thermal properties, with a lower glass transition temperature of −72 °C compared to BNDPA/F (−67 °C) and a higher thermal decomposition temperature of 179 °C compared to BuNENA (173 °C). The enthalpy of formation and heat of explosion of NFPEG3N3 were calculated to be −41.7 kJ mol^−1^ and 3421 J g^−1^, respectively. The impact of NFPEG3N3 on the glass transition temperature, viscosity and decomposition of the energetic binder glycidyl azide polymer (GAP)-diol was investigated and showed a remarkable decrease in viscosity (45.4%) and glass transition temperature (−3.3 °C) when compared to benchmark plasticizers in 10 wt% mixtures. These results demonstrate the potential of NFPEG3N3 as an insensitive and highly energetic plasticizer.

## Abstract 2

Novel nitrofurazanyl ethers have been synthesized, demonstrating that the heterocyclic moiety is an excellent building block for mechanically insensitive energetic plasticizers.

## Graphical abstract

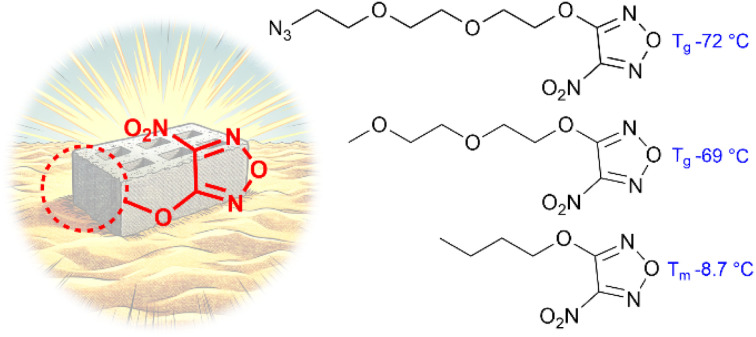

## Introduction

Energetic plasticizers are critical to the development of advanced solid propellants and polymer-bonded explosives.^1–4^ Typically, these plasticizers are high-boiling organic liquids that improve the mechanical properties and lower the glass transition temperature of binders. They also help reduce the viscosity during processing and can modify the burn rate of energetic formulations. Unlike inert plasticizers, energetic plasticizers contain energetic groups such as nitro, nitrate ester, nitramino, and azido, which increase the overall energy of the formulation. Depending on the application, the ideal properties for energetic plasticizers can vary and sometimes be contradictory. In general, they should have high heat of formation, high oxygen balance, low glass transition temperature, low migration tendency, low viscosity, high thermal stability and low mechanical sensitivity.^5^ Investigation of the glass transition temperature depression, viscosity reduction and decomposition temperature shift of a liquid plasticizer-binder mixture compared to pure binder samples provides initial insight into the suitability of a new compound.^6^

**Fig. 1:**
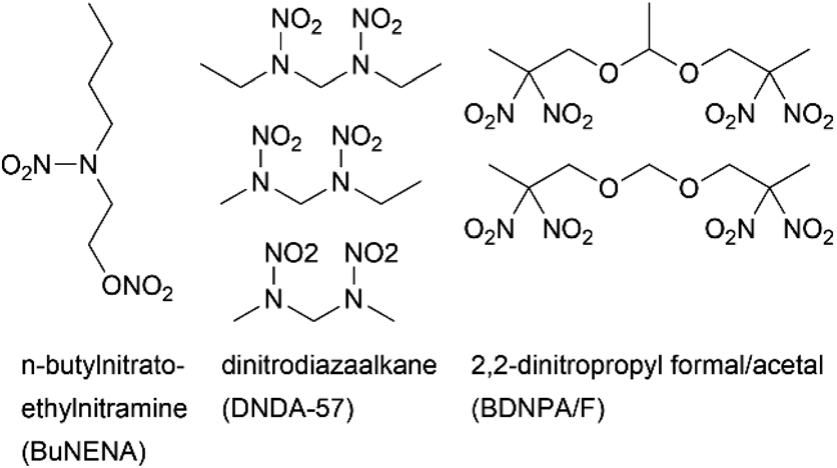
Examples of energetic plasticizers in use.

Known energetic plasticizers such as 2,2-dinitropropyl formal/acetal^7,8^ (BDNPA/F), *n*-butylnitratoethylnitramine^9^ (BuNENA) and dinitrodiazaalkane^10^ (DNDA-57) primarily consist of a saturated, linear carbon backbone, augmented by heteroatoms, most of which form energetic side groups (Fig. 1). Studies on heterocycle-based energetic plasticizers remain limited in the literature although heterocyclic building blocks are energy-rich and have been extensively studied for secondary and primary explosives.^11–13^

In particular, oxadiazoles may be advantageous for plasticizers due to their higher oxygen content compared to triazoles or tetrazoles. Among the oxadiazole isomers, the 1,2,5-oxadiazole (furazan) ring has the highest heat of formation at 216 kJ mol^−1^.^15–17^ Notably, 3,4-dinitrofurazan (DNF) is a liquid at room temperature, with a melting point of −15 °C.^18–20^ Although its synthesis is challenging, we have recently improved its safety by using a flow process.^21^ Derivatization of DNF *via* nucleophilic substitution is a straightforward reaction, as demonstrated by Sheremetev *et al.*^22,23^ In this work we present novel energetic plasticizers that exploit the outstanding energetic properties of the nitrofurazan structure.

## Results and discussion

### Synthesis

**Scheme 1:**
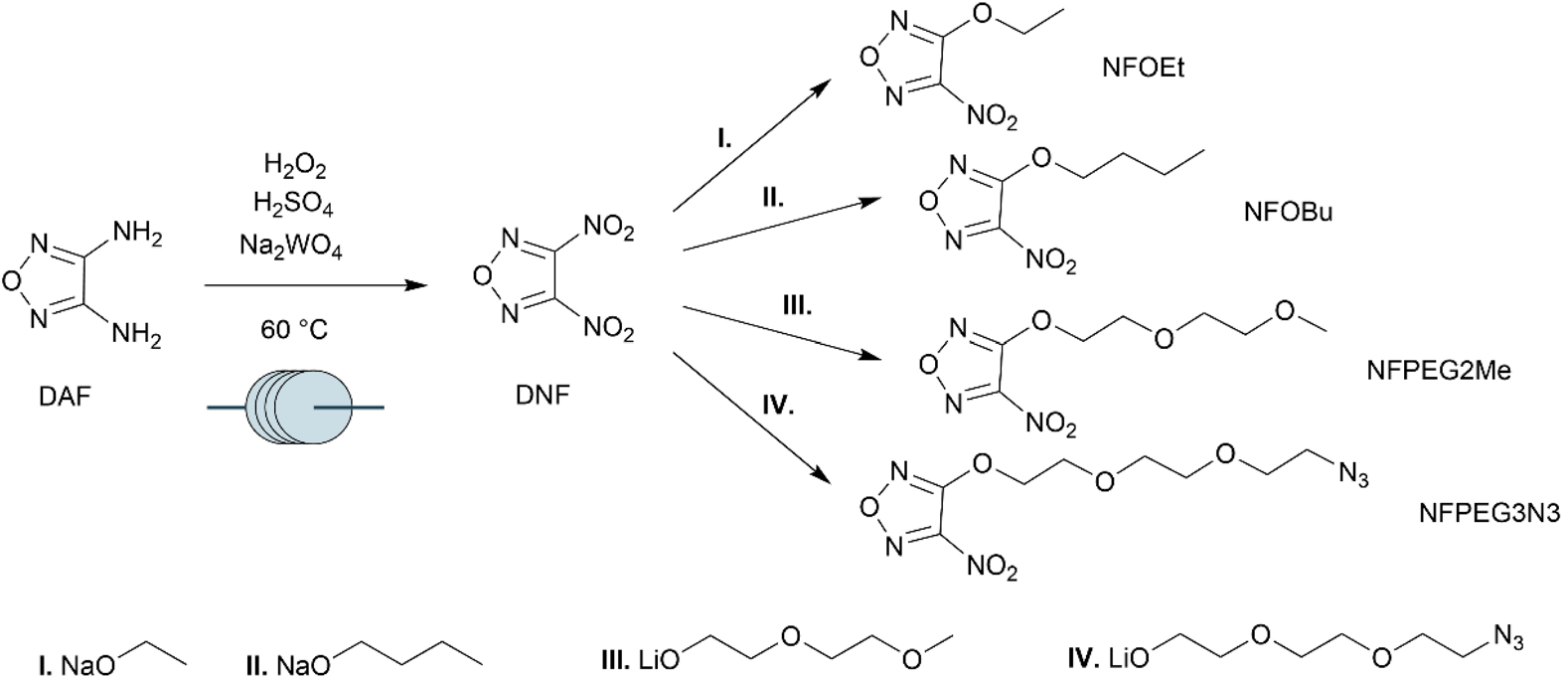
Synthesis of nitrofurazanyl ethers starting from DAF.

The starting material chosen for the synthesis was 3,4-diaminofurazan (DAF). Products were obtained by two-step syntheses (Scheme 1). First, DNF was obtained by complete amine oxidation of DAF with a mixture of hydrogen peroxide, sulfuric acid and sodium tungstate at 60 °C according to the flow chemistry procedure we reported.^21^ DNF is a sensitive primary explosive. However, in the context of the synthesis shown here, it can be safely handled and stored diluted in anhydrous dichloromethane solution. Side chains were introduced by nucleophilic substitution with sodium and lithium alkoxides, eliminating a nitro group as the corresponding nitrite. NFOEt was prepared based on literature methods.^22^ The purity of the longer-chain compounds NFPEG2Me and NFPEG3N3 was verified using HPLC (S6†). Non-commercially available alkoxides were synthesized by deprotonation of the alcohols with *n*-butyllithium in tetrahydrofuran. The deprotonation of the alcohols and the substitution reaction were performed at −94 °C. The prepared alkoxides were then used in the substitution reaction without further purification.

### Physicochemical properties

An overview of the properties of the synthesized nitrofurazanyl ethers is given in Table 1, in comparison to conventional energetic plasticizers. The heat of explosion and gas volume generated were determined using the ICT-thermodynamic code.^24^ The compounds show a heat of explosion ranging from 3004 J g^−1^ to 3976 J g^−1^ and a generated gas volume ranging from 928 cm^3^ g^−1^ to 956 cm^3^ g^−1^, which are similar to energetic plasticizers in use. However, significant differences are observed for the glass transition temperature.

**Table 1:** Comparison of conventional energetic plasticizers in use to synthesized nitrofurazanyl ethers

Compound	*T*_g_ a [°C]	*T*_m_ b [°C]	*T*_TGA_ c [°C]	*T*_dec_ d [°C]	IS^e^ [J]	*ρ* f [g cm^−3^]	*Q*_x_ g [J g^−1^]	OB_CO_2__^h^ [%]	N^i^ [%]	*V*_x_ j [cm^3^ g^−1^]
BDNPA/F^k^	−67		182	207	3	1.39	3469	−57.6	17.6	957
DNDA-57^k^	−52		159	221	3	1.35	3848	−72.3	30.9	1078
BuNENA^k^	−82		152	173	6	1.22	3573	−104.3	17.4	1045
NFOEt		8.7	65	233	>40	1.33	3976	−65.4	26.4	928
NFOBu		−6.2	88	191	>40	1.21	3379	−106.9	22.5	940
NFPEG2Me	−69		135	156	>40	1.30	3004	−92.6	18.2	956
NFPEG3N3	−72		167	179	>40	1.34	3421	−88.8	29.2	948

a Glass transition temperature measured by DSC.

b Melting point measured by DSC.

c Temperature at maximum mass loss rate measured by TGA.

d Thermal decomposition temperature (onset) measured by DSC in pressure-tight crucibles.

e Impact sensitivity measured by BAM drop hammer.

f Density.

g Heat of explosion calculated by ICT-thermodynamic code (water liquid).

h Oxygen balance calculated on CO_2_.

i Nitrogen content.

j Gas volume calculated by ICT-thermodynamic code without H_2_O at 25 °C.

k Physical properties of conventional energetic plasticizers partially from Schaller *et al.*^14^

**Fig. 2:**
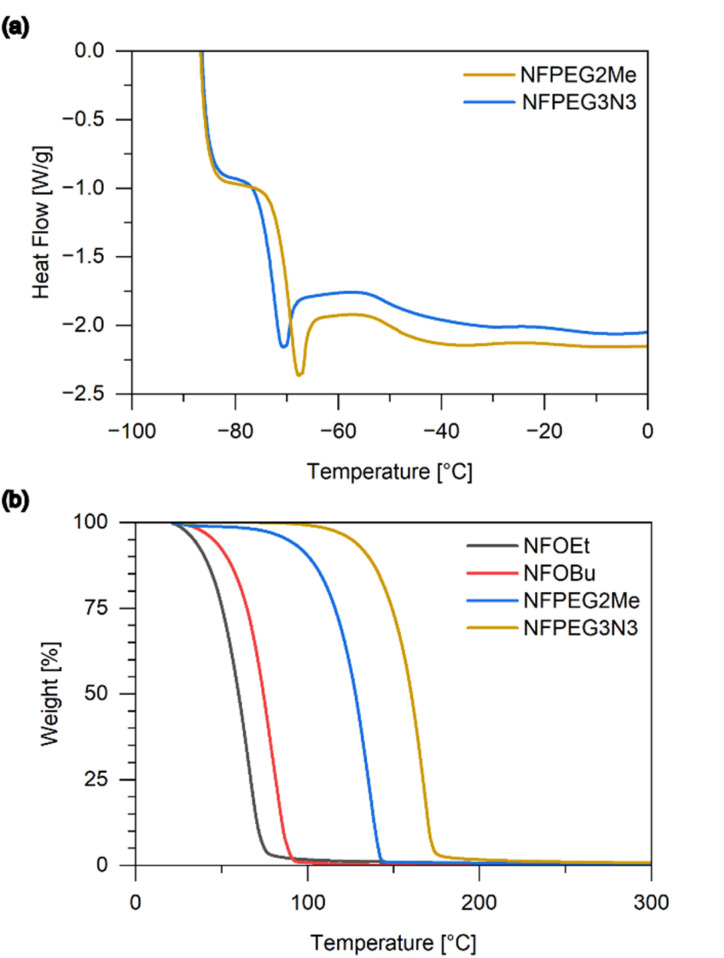
(a) Glass transition temperature curves obtained by DSC (b) thermal mass loss curves obtained by TGA.

The glass transition temperature of the ethylene glycol type side chain-containing ethers NFPEG2Me and NFPEG3N3 are −69 °C and −72 °C, respectively (Fig. 2a). These are slightly higher than the glass transition temperature of BuNENA (−82 °C), but lower than that of BNDPA/F (−67 °C). In contrast, NFOEt and NFOBu do not exhibit a glass transition, but rather melting points at 8.66 °C and −6.19 °C, respectively. The thermal stability of the compounds was analyzed by DSC in pressure-tight stainless steel crucibles and TGA under nitrogen flow. The alkyl ethers showed significantly higher thermal stability than the ethylene glycol ethers in DSC but evaporated at lower temperatures as shown in the TGA (Fig. 2b). The exothermic onsets for NFPEG2Me and NFPEG3N3 in DSC were found to be 156 °C and 179 °C, respectively. The complex DSC curves suggest a multi-stage decomposition processes (S4†). Until now we have no information about the exact mechanism of the decomposition of these substances. Compared to the reference plasticizers, NFPEG2Me shows a lower thermal stability while NFPEG3N3 outperforms BuNENA. These properties suggest that, depending on the side chain, the new materials could potentially be used as plasticizers in energetic formulations such as solid rocket propellants. They could also potentially be used as high-explosive plasticizers or in modern gun propellant formulations. The ethylene glycol type ethers have an oxygen balance slightly higher than BuNENA and NFPEG3N3 has a high nitrogen content of 29.2%, comparable to DNDA-57. In general, a compound with a higher oxygen balance tends to be more explosive, powerful, or sensitive.^25^

The densities of the synthesized compounds are between 1.30 g cm^−3^ and 1.34 g cm^−3^, except for NFOBu which has a much lower density of 1.21 g cm^3^. NFPEG3N3 has a density similar to that of GAP-diol (1.34 g cm^3^) which ensures a good miscibility. The impact sensitivity was determined by using standard BAM techniques.^26^ All nitrofurazanyl ethers have been found to be non-impact sensitive within UN regulations (>40 J). As a result, they exhibit superior mechanical sensitivity compared to conventional energetic plasticizers. NFPEG3N3 has a low viscosity of 32 mPa s at 20 °C, ensuring adequate processability over a wide temperature range.

### Infrared spectroscopy

**Fig. 3:**
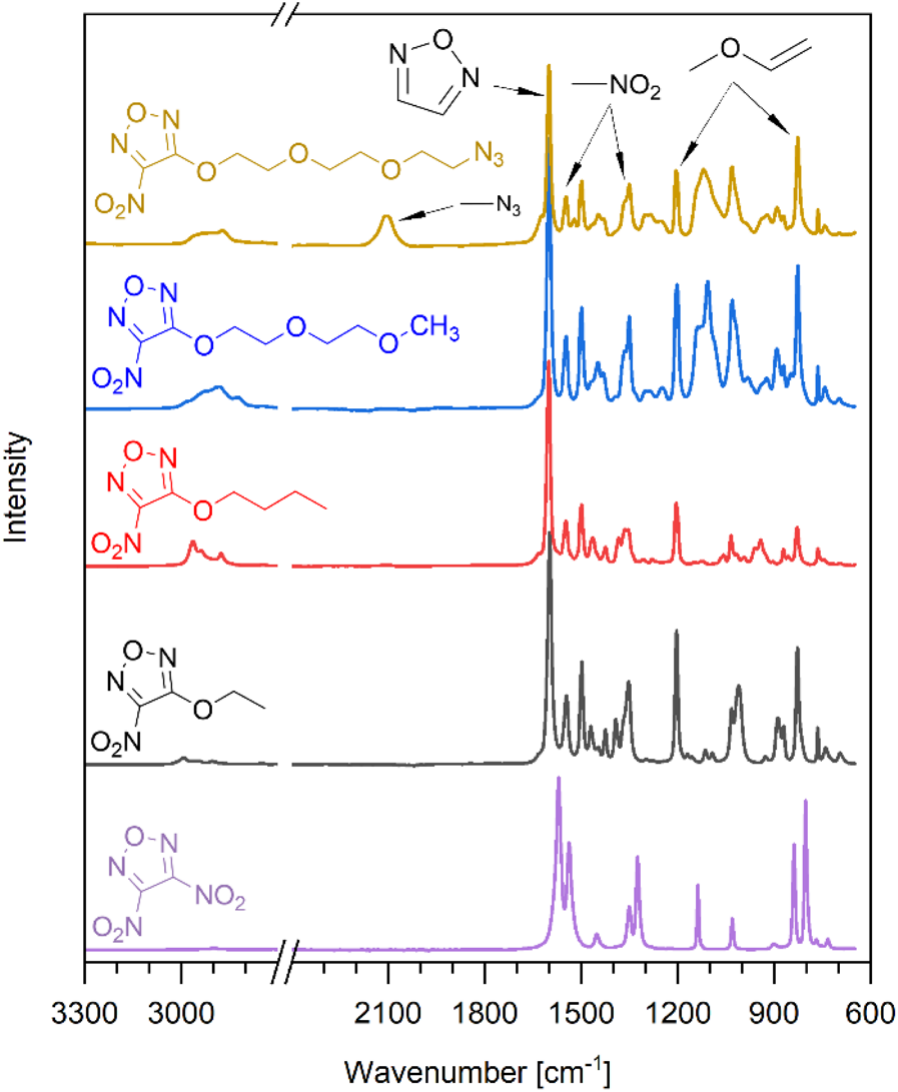
Infrared spectra of synthesized nitrofurazanyl ethers and 3,4-dinitrofurazan.

The infrared spectra of the nitrofurazanyl ethers and DNF were analyzed, and specific group frequencies were identified (Fig. 3).^27^ While the 1,2,5-oxadiazole ring vibration is found at 1570 cm^−1^ for DNF, it shifts to 1560 cm^−1^ for the ethers. The asymmetric stretching of the nitro group also shifts from 1538 cm^−1^ to 1548–1544 cm^−1^. In contrast the symmetric stretching of NO_2_ shows no shift and is found at 1350 cm^−1^ for all compounds. Specific frequencies for an allylic ether occur at 1203 cm^−1^ and 828 cm^−1^ indicating the replacement of a nitro group by the alkoxy substituents. The characteristic N=N stretching of the azide group is found for NFPEG3N3 at 2106 cm^−1^. The analysis shows that the IR spectroscopy allows rapid identification and reaction control in this case.

### Calculation of the formation enthalpy

The standard enthalpy of formation of the synthesized nitrofurazanyl ethers in liquid-state was calculated from the gas-phase enthalpy of formation and the vaporization enthalpy using density functional theory calculations within the Gaussian 16 software package.^28^ The vaporization enthalpies were estimated using the semi-empirical methods of Politzer,^29^ Rice^30^ and improved Rice^31^ (Table 2). In our experience, for molecules without any enthalpy data in literature, an average of all three methods gives the best results as it provides the broadest empirical base. The methods are based on geometry optimizations and calculations of the ground state energy with the DFT functional/basis set combinations B3PW91/6-31G** and B3LYP/6-31G*. The improved method of Rice utilizes B3LYP/6-311++G(2df,2p) for calculation of the electronic energy of the ground state.

**Table 2:** Vaporization enthalpies calculated by the methods of Rice and Politzer

Δ*H*^∅^_vap._ [kJ mol^−1^]	Politzer	Rice	Rice II	Average
NFOEt	53.5	58.5	62.6	58.2
NFOBu	60.8	68.7	74.9	68.1
NFPEG2Me	69.7	80.5	88.4	79.6
NFPEG3N3	78.6	92.5	103.1	91.4

**Fig. 4:**
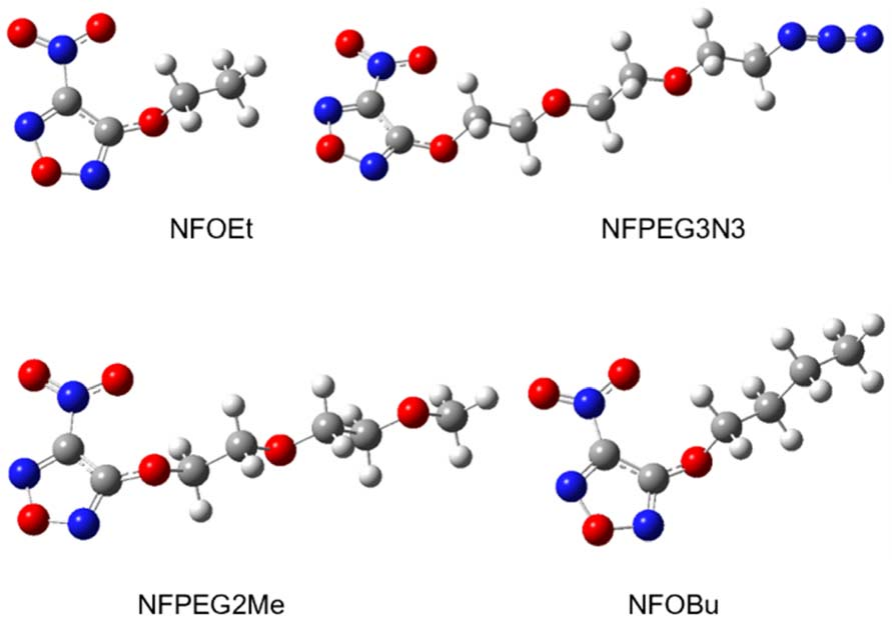
Molecular structures of the synthesized nitrofurazanyl ethers geometry optimized using the G4 composite method.

The functional/basis set combinations B3PW91/aug-cc-pVTZ and B3LYP/aug-cc-pVTZ were also used to find and validate the energetic ground state. Since the accuracy of the DFT methods is not sufficient for the calculations of the gas phase enthalpy of formation with the atomization method, geometry optimizations and ground state energy calculations were performed with the so-called composite methods CBS-QB3,^32,33^ G4 (ref. 34 and 35) and G4MP2 (ref. 35) (Table 3 and Fig. 4). Finally, the standard enthalpy of formation of the liquid phase was determined by combining results of the precise G4 calculations and average value of the three semi-empirical values for the vaporization enthalpy (Table 4). The standard enthalpy of formation of the liquid state for NFOEt, NFOBu, NFPEG2Me, and NFPEG3N3 were found to be −13.8 kJ mol^−1^, −72.7 kJ mol^−1^, −339.5 kJ mol^−1^, and −41.7 kJ mol^−1^, respectively.

**Table 3:** Gas-phase heat of formation calculated by composite methods

Δ_f_*H*^∅^_m_ [kJ mol^−1^]		CBS-QB3	G4		G4MP2
NFOEt	29.2		44.4	56.7	
NFOBu	−17.7		−4.5	7.7	
NFPEG2Me	−285.2		−260.0	−244.0	
NFPEG3N3	26.8		49.7	70.0	

**Table 4:** Standard heat of formation in the liquid phase

	Δ_f_*H*^∅^_m_(l) [kJ mol^−1^]	Δ_f_*H*^∅^_m_(l) [kcal mol^−1^]
NFOEt	−13.8	−3.31
NFOBu	−72.7	−17.36
NFPEG2Me	−339.5	−81.14
NFPEG3N3	−41.7	−9.98

### Impact of NFPEG3N3 in mixtures with GAP-diol

**Fig. 5:**
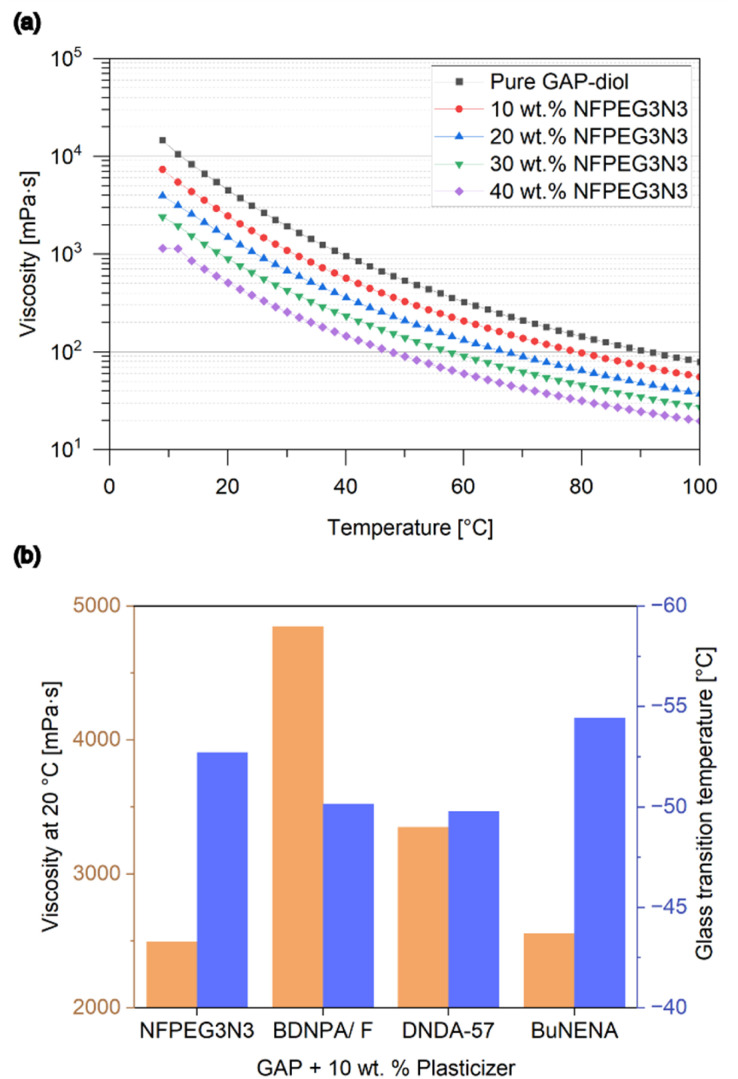
(a) Logarithmic representation of temperature and NFPEG3N3 concentration impact on the viscosity of GAP-diol. (b) Viscosity and glass transition temperature of GAP-diol with 10 wt% of NFPEG3N3 and selected plasticizers in use.

In addition to the mechanical properties of the cured formulation, plasticizers are also critical to the processability of the uncured formulation. Therefore, we studied the viscosity change of mixtures of GAP-diol and NFPEG3N3 with ratios from pure GAP-diol to 40 wt% at 10 °C to 100 °C (Fig. 5a).

**Fig. 6:**
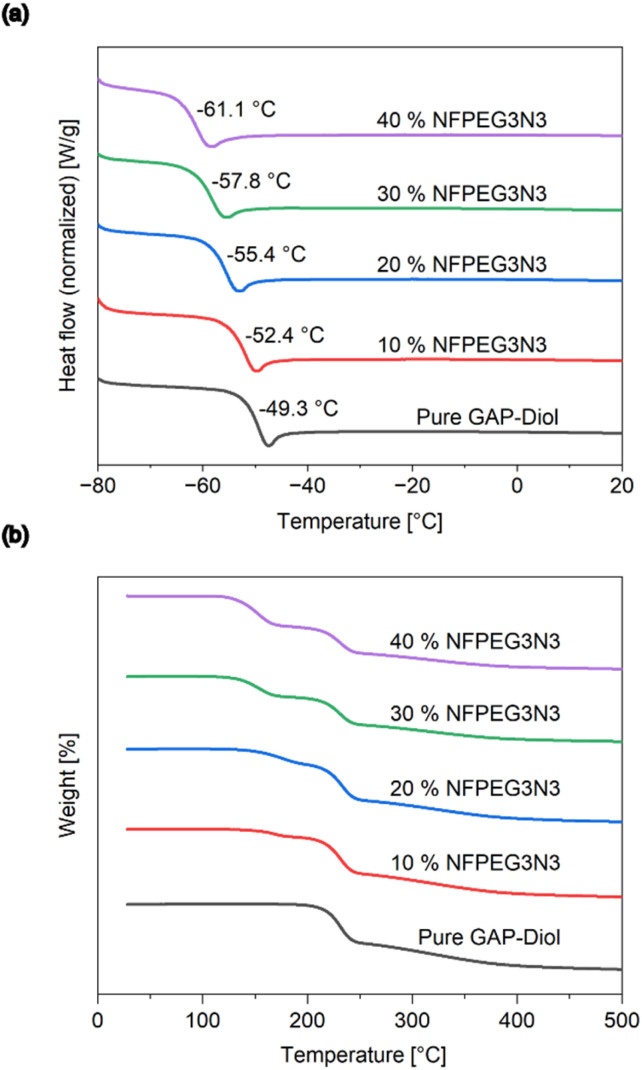
(a) Glass transition temperatures of pure GAP-diol and mixtures with NFPEG3N3 obtained by DSC. (b) TGA-curves of pure GAP-diol and mixtures with NFPEG3N3.

Newtonian behavior was observed for all measured compounds and mixtures. With increasing plasticizer concentration, the viscosity decreases at all measured temperatures, but each increase in plasticizer concentration has a smaller effect than the previous one. In direct comparison with other plasticizers at 10 wt% NFPEG3N3 shows a superior viscosity reduction of GAP-diol from 4560 mPa s to 2490 mPa s at 20 °C. This result is slightly better than BuNENA, which reduced the viscosity of GAP-diol to 2560 mPa s in our measurement (Fig. 5b). NFPEG3N3 also shows a competitive lowering of the glass transition temperature from −49.4 °C to −52.7 °C. At concentrations up to 40 wt% a linear decrease of the glass transition temperature compared to pure GAP-diol was observed (Fig. 6a). First results on the compatibility of GAP and NFPEG3N3 could be received by TGA measurements of mixtures and pure substances (Fig. 6b). Two separate decomposition peaks were found for all investigated mixtures. The first decomposition event can be attributed to NFPEG3N3 which shows a mass loss in TGA at 167 °C as a pure substance and in a 10 wt% mixture in GAP-diol. The second decomposition event could be attributed to the GAP diol and took place constantly at 231 °C without any influence of the presence or content of NFPEG3N3.

## Conclusion

In this study, three new nitrofurazanyl ethers were synthesized in high yields. Theoretical calculations show that nitrofurazanyl ethers with ethylene glycol-type side chains have promising energetic properties, including high heat of explosion and large generated gas volume. All synthesized ethers were found to be non-impact sensitive within UN regulations.^26^ Among the synthesized compounds, NFPEG3N3 emerged as the most promising candidate for use as an energetic plasticizer. Remarkably, NFPEG3N3 outshines BuNENA with its superior oxygen balance, nitrogen content and decomposition temperature, while maintaining a low glass transition temperature of −72 °C and viscosity of 32 mPa s at 20 °C. In addition, NFPEG3N3 demonstrated a significant ability to reduce viscosity and glass transition temperature when formulated with GAP diol. NFPEG3N3 does not adversely affect the decomposition temperature of GAP-diol, even at concentrations up to 40 wt%. These results demonstrate for the first time the potential of nitrofurazanyl ethylene glycol ethers as advanced energetic plasticizers.

## Experimental

### Materials and instruments

BuNENA and BDNPA/F were purchased from Chemring. DNDA-57 was obtained from N. D. Zelinsky Institute of Organic Chemistry (Moscow, Russia). DAF was purchased from Chemicalpoint (Deisenhofen, Germany). 2-(2-(2-Azidoethoxy)ethoxy)ethan-1-ol (PEG3N3) was purchased from Abcr (Karlsruhe, Germany). GAP-diol charge 03S19 (*M*_W_ 1814 g mol^−1^, EQ 1228 g mol^−1^, functionality 1.5) was supplied from Eurenco. Other chemicals were purchased from Sigma Aldrich, Merck or Carl Roth. Unless otherwise stated, all chemicals were used without further purification. A cooling bath from liquid nitrogen and acetone was used for low temperature reactions. ^1^H-NMR and ^13^C-NMR spectra were recorded on a 400 MHz Bruker AV-400 spectrometer. The melting point and glass transition temperature were recorded on a TA instruments Q1000 differential scanning calorimeter (DSC) at a heat rate of 10 °C min^−1^ using pierced aluminum crucibles. Glass transition temperatures were measured from heating up after cooling to −90 °C. Reported glass transition temperatures are inflection point temperatures. Reported melting points are peak temperatures. Measurements were carried out under nitrogen flux (25 mL min^−1^). The thermal decomposition temperature was measured on the same apparatus at a heat rate of 5 °C min^−1^ in pressure-tight steel crucibles (F20) purchased from the Swiss Institute for the Promotion of Safety. Thermogravimetric analysis (TGA) was performed on a TA Q500 apparatus with a heating rate of 5 °C min^−1^ in a platinum 100 μL pan under nitrogen flux (25 mL min^−1^). Reported values are the central points, according to DIN ENISO 11358. Infrared (IR) spectra were recorded on a Thermo Scientific iS 50 FTIR spectrometer in attenuated total reflection (ATR) mode. Raman spectra were recorded on a Bruker FT MultiRAM spectrometer at 1064 nm (Neodymium-YAG laser). Densities were determined by scale and volumetric flask. Elemental analyses were performed on a Thermo Flash EA with helium as carrier gas. Carbon, hydrogen and nitrogen were determined at a combustion temperature of 900 °C using a tin sleeve and oxygen at 1090 °C using a silver sleeve. High resolution mass spectra were recorded on a Thermo Fisher QExactive Plus spectrometer using electrospray ionization (ESI). Mechanical sensitivity was measured on a BAM drop hammer. A high-pressure liquid chromatography system Agilent 1200 equipped with a ZorbaxBonus RP 4.6 × 2500 mm column and a diode array detector was used to verify the purity of NFPEG2Me and NFPEG3N3. Gradient determinations were performed by injecting 5 μL acetonitrile diluted samples of the products. The mobile phase was acetonitrile/water with 0.1% trifluoroacetic acid (gradient from 20 : 80 to pure acetonitrile in 15 minutes) the flow rate was 1.0 mL min^−1^.

### Quantum and thermochemical calculations

Quantum chemical calculations were performed with the software Gaussian 16.^28^ The molecular structures were calculated as singlets in their electronic ground states and verified as true minima by frequency calculations (no imaginary frequencies). The electrostatic potential of the optimized structures was investigated using the software MultiFWN.^36,37^ Thermochemical data were calculated using ICT-thermodynamic code.^24^

### General procedure for the preparation of nitrofurazanyl ethers

#### General procedure 1: preparation of the alkoxide solution

To a solution of the alcohol in THF, 2.5 M *n*-butyllithium in hexane was added to that at −94 °C and under protective atmosphere. The reaction mixture was slowly warmed up to ambient temperature. The alkoxide solution was subsequently used for the substitution without further purification.

#### General procedure 2: substitution

To a solution of DNF in DCM, the alkoxide solution was added to that at −94 °C under protective atmosphere. The reaction mixture was slowly warmed up to ambient temperature. Then it was washed with water (2 × 300 mL) and the organic phase was dried over Na_2_SO_4_. The solvent was removed, and the residue was purified by basic alumina column chromatography using 25 vol% ethyl acetate–petroleum ether to deliver a pure product.

#### Synthesis of 3,4-dinitro-1,2,5-oxadiazole (DNF)

DNF was prepared according to the literature.^21^ It was received as a colorless solution in dichloromethane (21.74 g L^−1^, 50%). IR (ATR, cm^−1^): 1570, 1538, 1451, 1351, 1325, 1137, 1031, 902, 839, 803, 769, 734, 615, 475. ^13^C NMR (400 MHz, CDCl_3_) *δ* [ppm] = 152.72 (t, *J* = 20.2 Hz).

#### Synthesis of 3-ethoxy-4-nitro-1,2,5-oxadiazole (NFOEt)

NFOEt (440 mg, 73%) was prepared according to the literature and received as a colorless oil.^22^ Mp 7.1 °C. TGA 65 °C. *T*_dec_ 233 °C. Found: C, 30.15; H, 3.25; N, 26.4; O, 40.2%; M^−^ (mass spectrum), 159.0275. C_4_H_5_N_3_O_4_ requires C, 30.2; H, 3.2; N, 26.4; O, 40.2%; M^−^, 159.0280. IR (ATR, cm^−1^): 2992, 1597 (furazan ring), 1545, 1498, 1471, 1446, 1425, 1393, 1353, 1298, 1204, 1170, 1156, 1114, 1093, 1033, 1011, 928, 889, 871, 828, 765, 741, 696, 593. Raman (1064 nm, cm^−1^): 2991, 2945, 2898, 2877, 2768, 2721, 1728, 1605, 1550, 1501, 1454, 1426, 1369, 1355, 1279, 1207, 1116, 1094, 1016, 928, 872, 831, 767, 742, 696, 598, 448. ^1^H NMR (400 MHz, CDCl_3_) *δ* [ppm] = 4.55 (q, *J* = 7.1 Hz, 2H), 1.55 (t, *J* = 7.1 Hz, 3H) ppm. ^13^C NMR (101 MHz, CDCl_3_) *δ* [ppm] = 158.19, 151.62 (t, *J* = 17.5 Hz), 70.80, 14.29. Impact sensitivity > 40 J. Friction sensitivity > 360 N. *ρ* 1.33 ± 0.02 g cm^−3^.

#### Synthesis of 3-butoxy-4-nitro-1,2,5-oxadiazole (NFOBu)

The reaction has been performed by following general procedure 2.

20 wt% sodium butoxide in butanol (10.65 g, 22.11 mmol) and DNF (3.37 g, 21.06 mmol) in dichloromethane (180 mL) were used. The product was received as colorless oil (3.11 g, 79%). Mp −7.2 °C. TGA 86 °C. *T*_dec_ 191 °C. Found: C, 38.5; H, 4.9; N, 22.3; O, 34.1%; M^−^ (mass spectrum), 187.0591. C_6_H_9_N_3_O_4_ requires C, 38.5; H, 4.85; N, 22.45; O, 34.2%; M^−^, 187.0593. IR (ATR, cm^−1^): 2964, 2938, 2877, 1598 (furazan ring), 1546, 1498, 1464, 1424, 1383, 1353, 1203, 1057, 1034, 1017, 994, 960, 942, 872, 857, 827, 765, 746, 592, 496. Raman (1064 nm, cm^−1^): 2940, 2918, 2877, 2743, 1603, 1550, 1500, 1453, 1426, 1356, 1303, 1263, 1232, 1204, 1147, 1127, 1059, 1036, 994, 961, 944, 906, 871, 832, 766, 696, 596, 438. ^1^H NMR (400 MHz, CDCl_3_) *δ* [ppm] = 4.48 (t, *J* = 6.5 Hz, 2H), 1.94–1.81 (m, 2H), 1.59–1.43 (m, 2H), 0.99 (t, *J* = 7.4 Hz, 3H). ^13^C NMR (101 MHz, CDCl_3_) *δ* [ppm] = 158.39, 151.63, 74.59, 30.61, 18.90, 13.68. Impact sensitivity > 40 J. Friction sensitivity > 360 N. *ρ* 1.21 ± 0.02 g cm^−3^.

#### Synthesis of 3-(2-(2-methoxyethoxy)ethoxy)-4-nitro-1,2,5-oxadiazole (NFPEG2Me)

The reaction has been performed by following the general procedures 1 and 2.

Diethylene glycol monomethyl ether (1.44 mL, 1.47 g,12.24 mmol), THF (5 mL), 2.5 M *n*-butyllithium in hexane (5 mL, 12.48 mmol) and DNF (1.96 g, 12.24 mmol) in DCM (90 mL) were used. The product was received as pale yellow liquid (2.31 g, 81%). *T*_g_ −69.2 °C. TGA 135 °C. *T*_dec_ 156 °C. Found: C, 34.95; H, 4.8; N, 17.9; O, 41.2%; [M + H]^+^ (mass spectrum), 234.0710. C_7_H_11_N_3_O_6_ requires C, 36.1; H, 4.8; N, 18.0; O, 41.2%; [M + H]^+^, 234.0726. IR (ATR, cm^−1^): 2883, 1599 (furazan ring), 1547, 1499, 1448, 1432, 1351, 1300, 1248, 1202, 1107, 1031, 983, 924, 892, 872, 828, 765, 743, 698, 591, 497. Raman (1064 nm, cm^−1^): 2947, 2895, 2830, 2742, 1604, 1550, 1501, 1472, 1446, 1431, 1368, 1287, 1243, 1205, 1132, 1032, 926, 872, 831, 766, 743, 699, 594, 441. ^1^H NMR (400 MHz, CDCl3) *δ* [ppm] = 4.63 (ddd, *J* = 5.8, 3.3, 1.2 Hz, 2H), 3.93 (ddd, *J* = 5.8, 3.4, 1.3 Hz, 2H), 3.74–3.67 (m, 2H), 3.57–3.51 (m, 2H), 3.36 (d, *J* = 1.2 Hz, 3H). ^13^C NMR (101 MHz, CDCl3) *δ* [ppm] = 158.40, 151.58, 73.75, 72.00, 71.03, 68.63, 59.16 ppm. Impact sensitivity > 40 J. Friction sensitivity > 360 N *ρ* 1.30 ± 0.02 g cm^−3^.

#### Synthesis of 3-(2-(2-(2-azidoethoxy)ethoxy)ethoxy)-4-nitro-1,2,5-oxadiazole (NFPEG3N3)

The reaction has been performed by following the general procedures 1 and 2.

2-(2-(2-Azidoethoxy)ethoxy)ethan-1-ol (2.65 g, 15.15 mmol), THF (10 mL), 2.5 M *n*-butyllithium in hexane (6 mL, 15.2 mmol) and DNF (2.42 g, 15.15 mmol) in DCM (140 mL) were used. The product was received as yellow liquid (3.50 g, 80%). *T*_g_ −72.4 °C. TGA 167 °C. *T*_dec_ 179 °C. Found: C, 32.9; H, 4.2; N, 28.0; O, 33.45%; [M + H]^+^ (mass spectrum), 289.0882. C_8_H_12_N_6_O_6_ requires C, 33.3; H, 4.2; N, 29.2; O, 33.3%; [M + H]^+^, 289.0897. IR (ATR, cm^−1^): 2872, 2100 (–N_3_), 1599 (furazan ring), 1547, 1499, 1446, 1351, 1284, 1205, 1119, 1032, 923, 891, 871, 852, 827, 765, 743, 697, 645, 591, 557, 505. Raman (1064 nm, cm^−1^): 2944, 2875, 2096, 1604, 1549, 1501, 1472, 1446, 1430, 1368, 1286, 1247, 1126, 1033, 992, 923, 872, 831, 165, 742, 699, 645, 593, 440. ^1^H NMR (400 MHz, CDCl_3_) *δ* [ppm] = 4.61–4.54 (m, 2H), 3.93–3.86 (m, 2H), 3.71–3.64 (m, 2H), 3.68–3.58 (m, 4H), 3.31 (dq, *J* = 4.7, 2.4 Hz, 2H). ^13^C NMR (101 MHz, CDCl_3_) *δ* [ppm] = 158.29, 73.62, 71.03, 70.72, 70.17, 68.58, 50.69. Impact sensitivity > 40 J. Friction sensitivity > 360 N. *ρ* 1.34 ± 0.02 g cm^−3^.

## Data availability

The data supporting this article, including infrared spectra, NMR spectra, mass spectra, DSC curves and HPLC chromatographs have been included as part of the ESI (S1–S6†).

## Conflicts of interest

There are no conflicts to declare.

## Supplementary Material

RA-015-D5RA01282A-s001
