# Supplementary material for: Synthesis and characterization of novel nitrofurazanyl ethers as potential energetic plasticizers
Source: RSC Adv. 2025 Apr 22;15(16):12577–84. doi: 10.1039/d5ra01282a (PMC12012608; doi:10.1039/d5ra01282a)
Supplement: RA-015-D5RA01282A-s001 [file RA-015-D5RA01282A-s001.pdf]

Supplementary Information (SI) for RSC Advances.
This journal is © The Royal Society of Chemistry 2025

Supplementary Information

^{1}H and ^{13}C NMR spectra (S1)

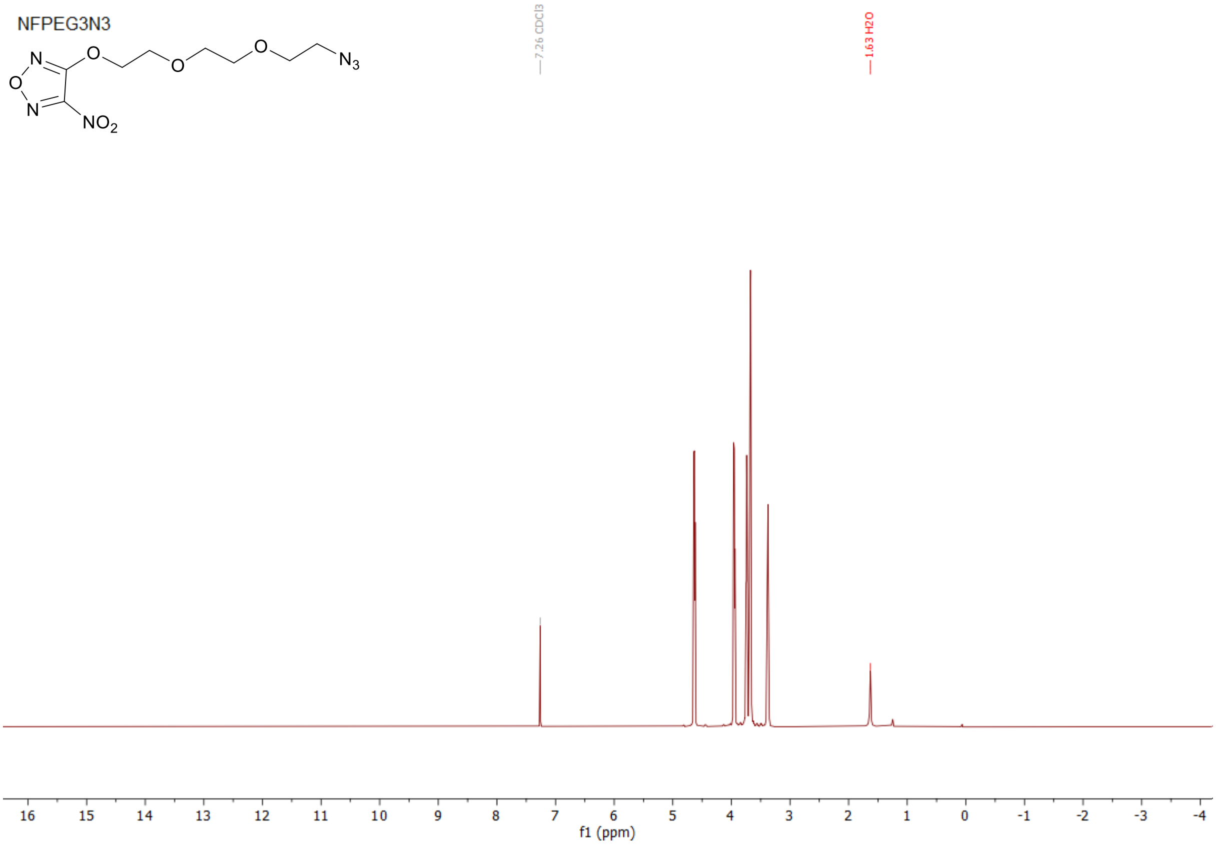

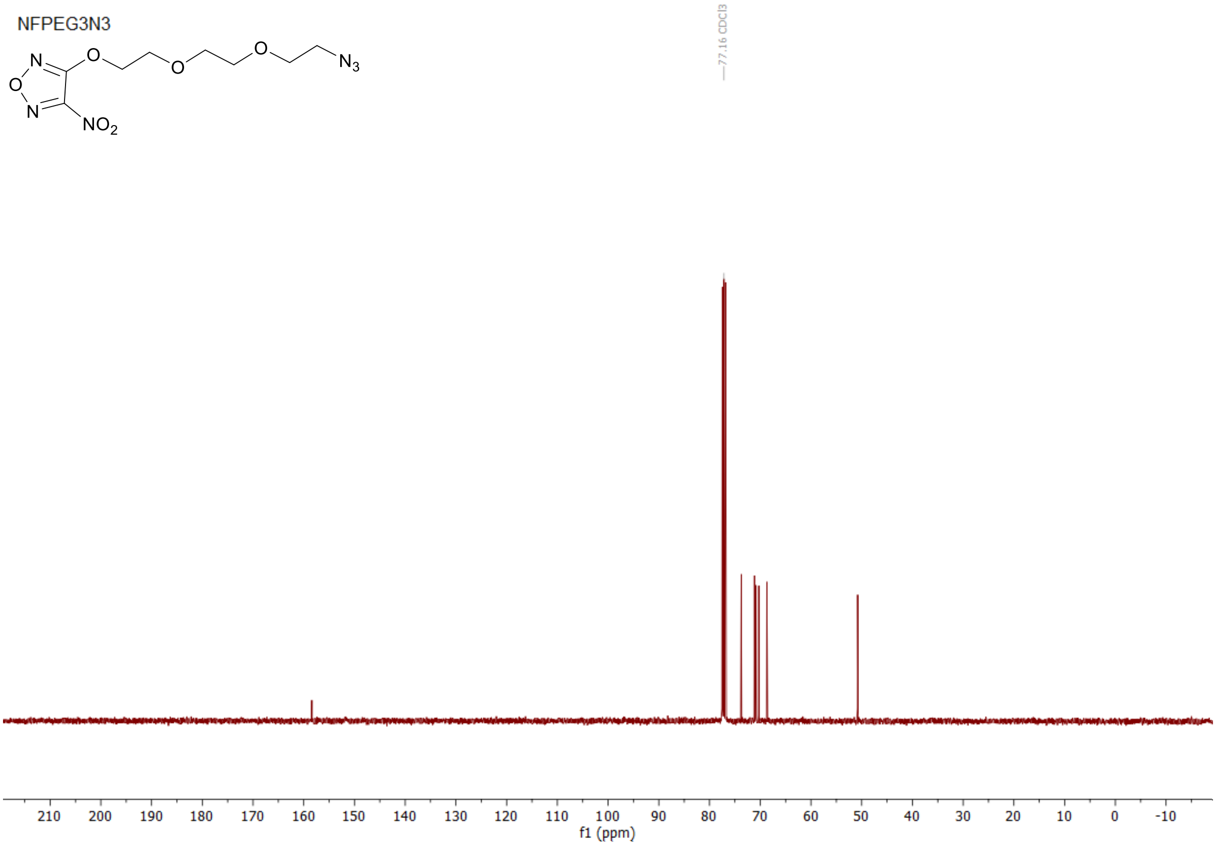

NFPEG2Me

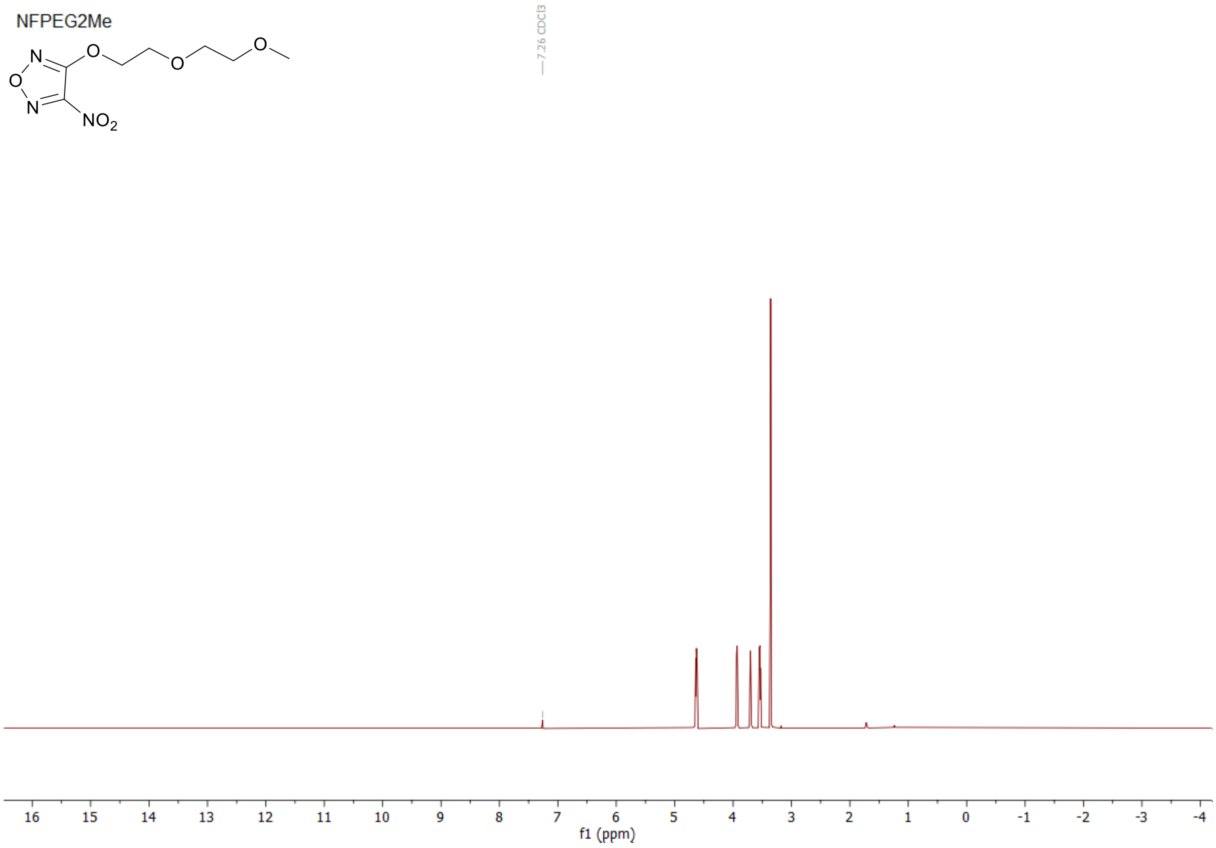

NFPEG2Me

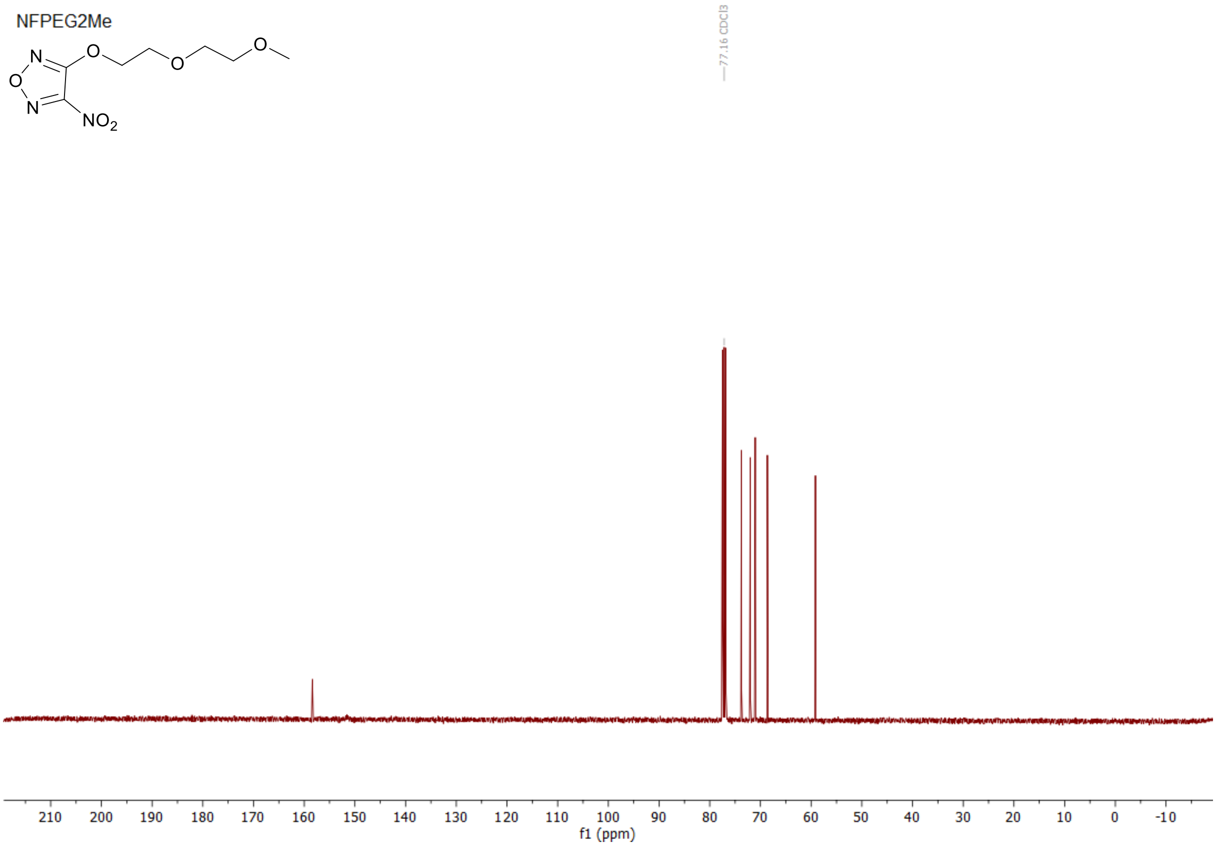

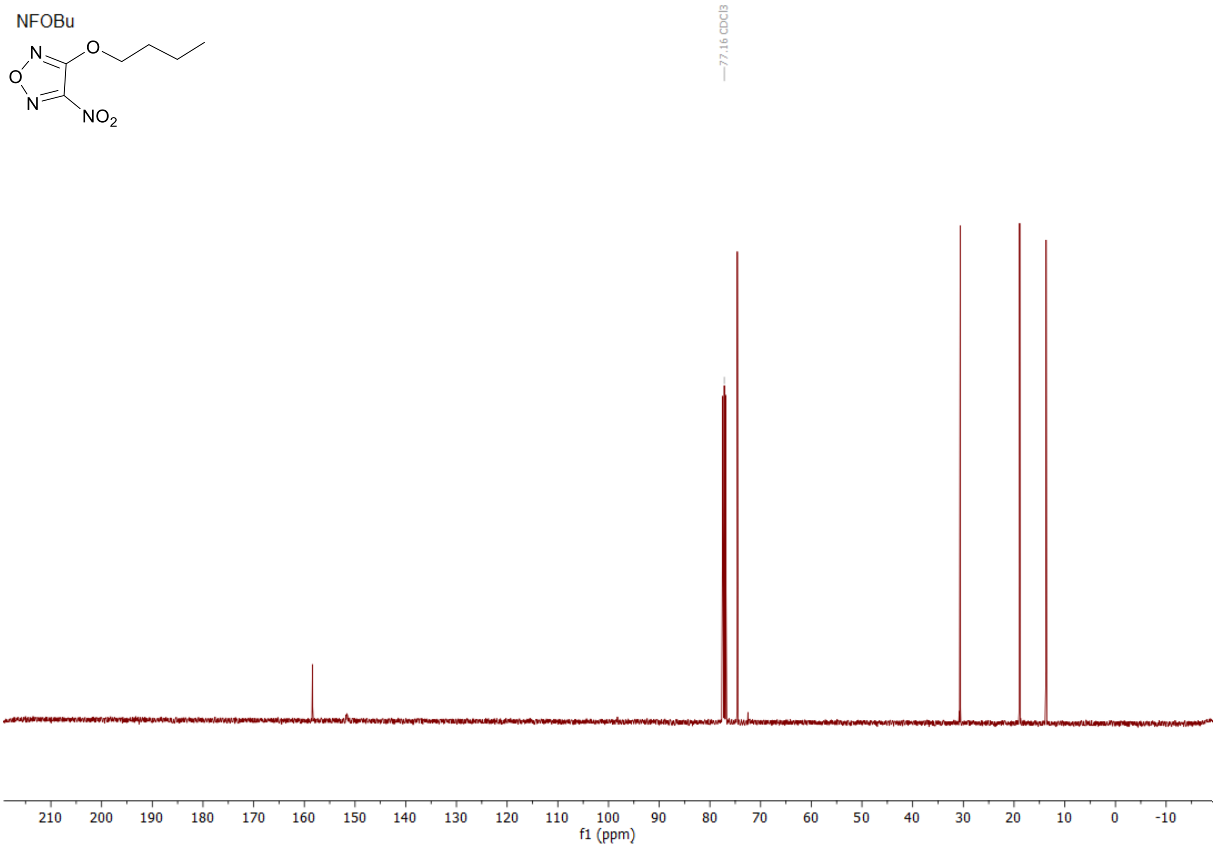
NFOBu
N
O
N
O
NO2
77.16 CDCl3
210 200 190 180 170 160 150 140 130 120 110 100 90 80 70 60 50 40 30 20 10 0 -10
f1 (ppm)

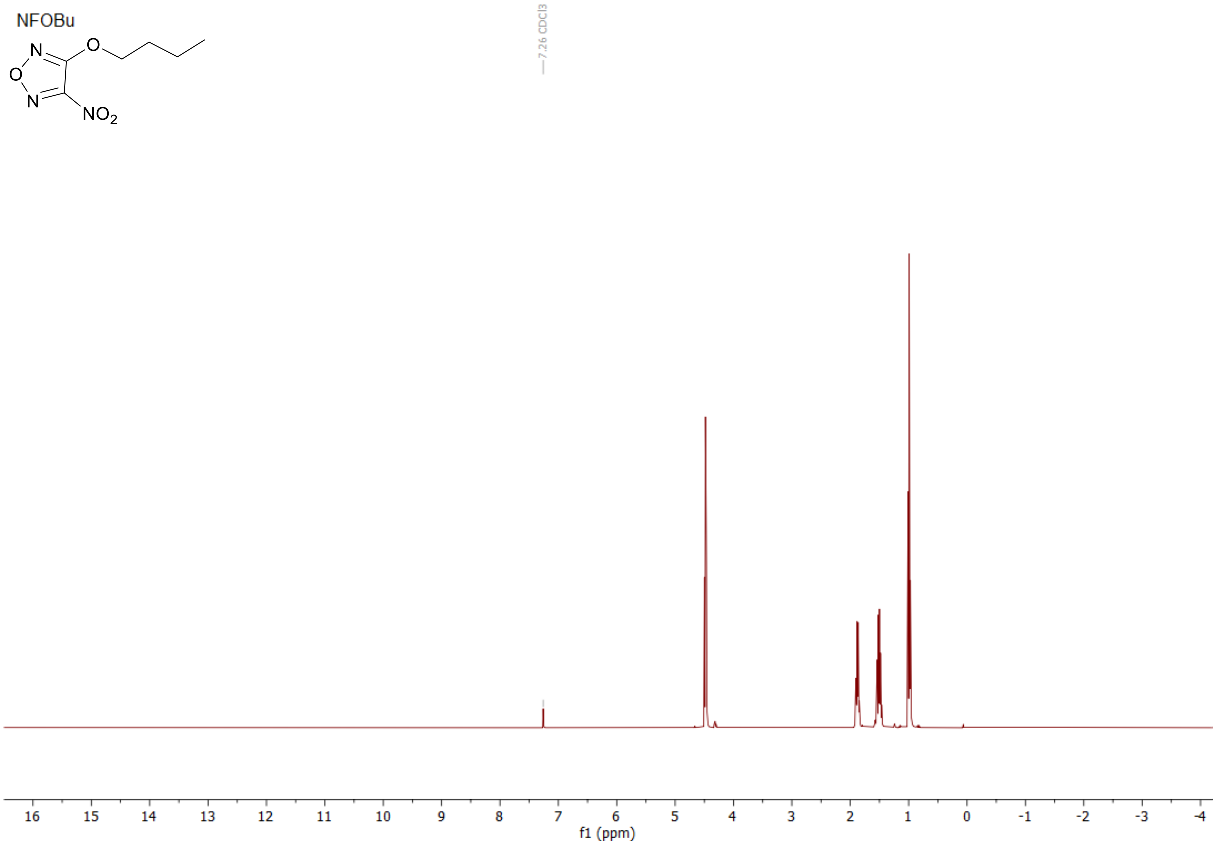
NFOBu
N
O
N
O
NO2
7.26 CDCl3
16 15 14 13 12 11 10 9 8 7 6 5 4 3 2 1 0 -1 -2 -3 -4
f1 (ppm)

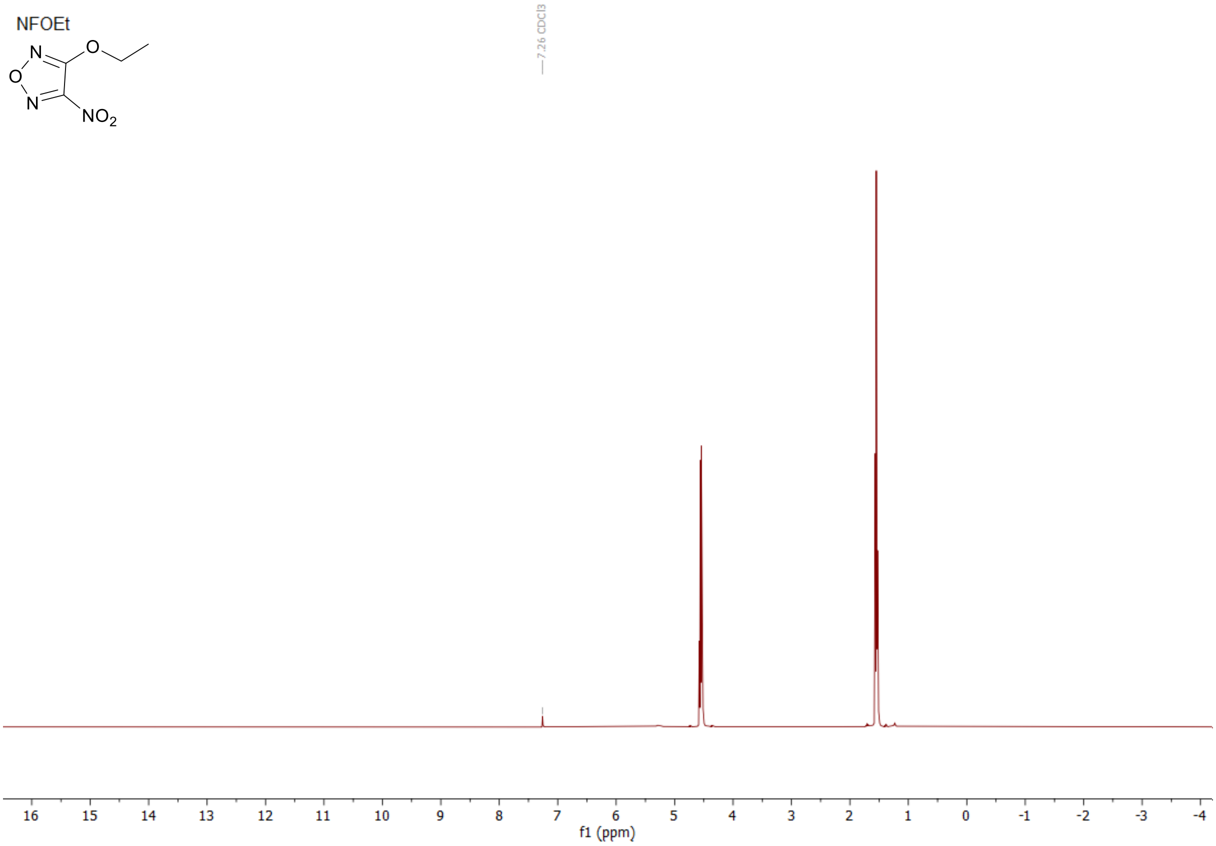
NFOEt
NO_2
7.26 CDCl3
16 15 14 13 12 11 10 9 8 7 6 5 4 3 2 1 0 -1 -2 -3 -4
f1 (ppm)

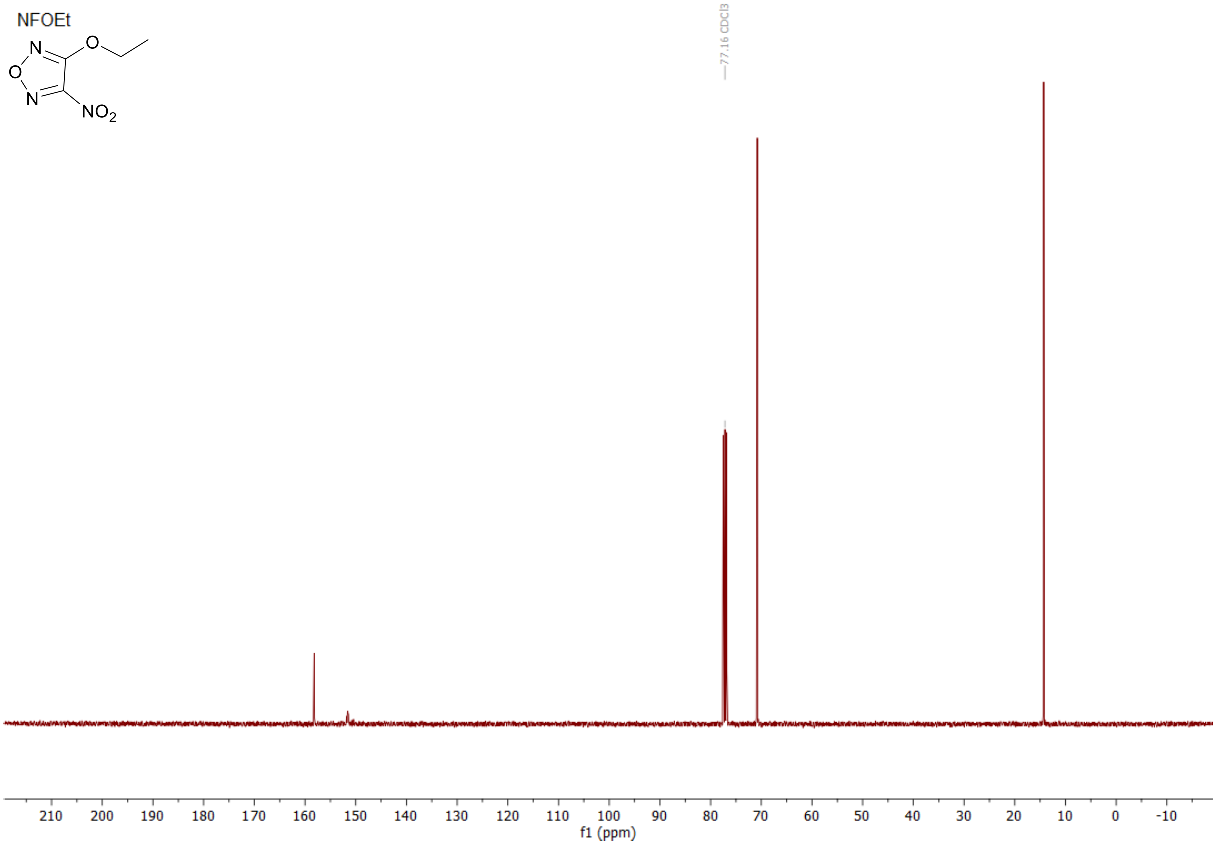
NFOEt
NO_2
77.16 CDCl3
210 200 190 180 170 160 150 140 130 120 110 100 90 80 70 60 50 40 30 20 10 0 -10
f1 (ppm)

Infrared spectra (S2)

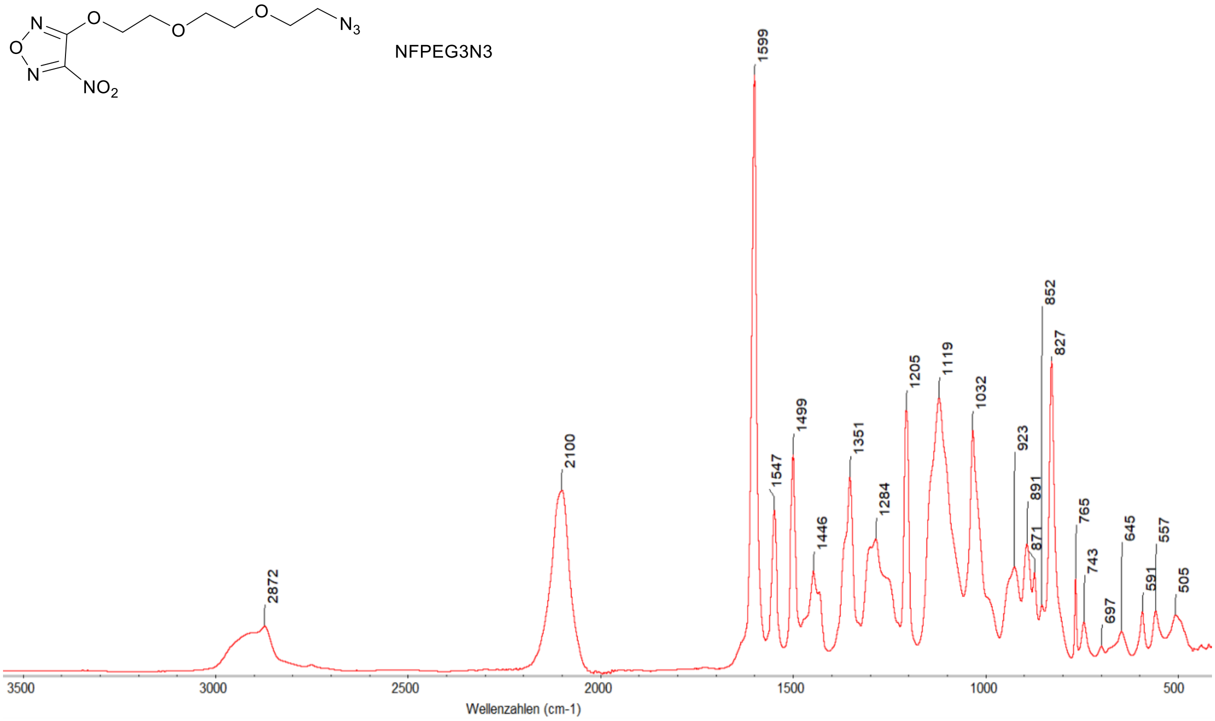

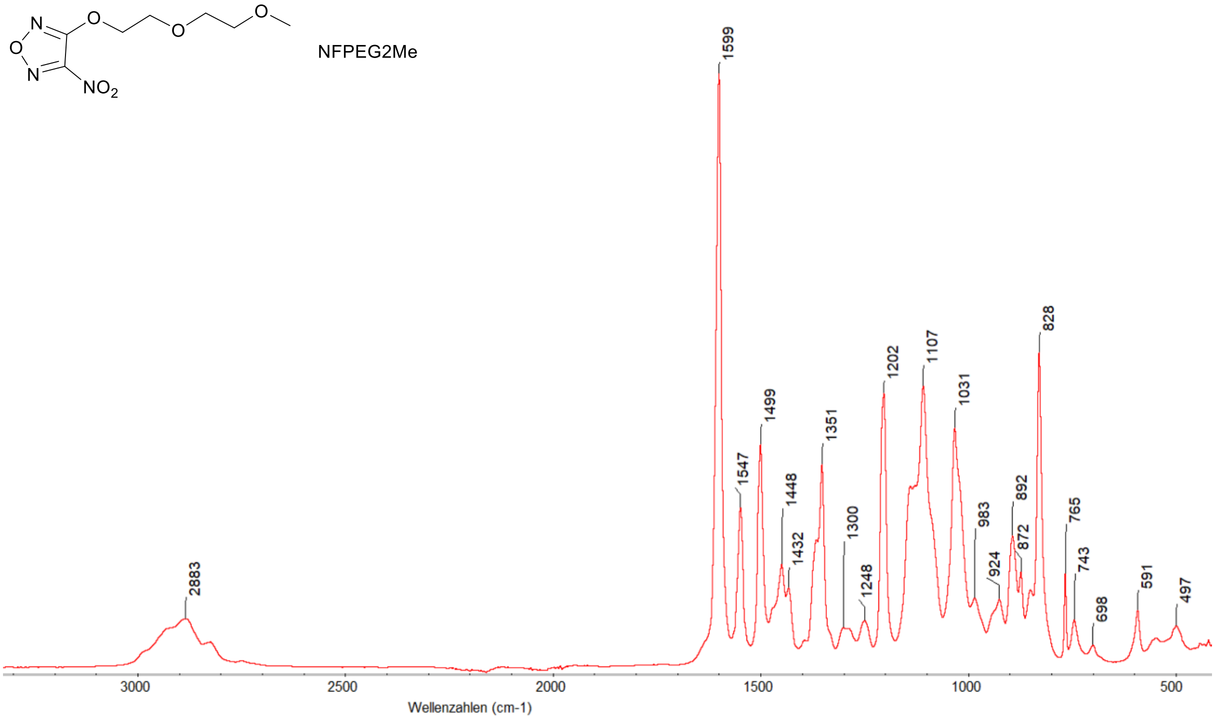

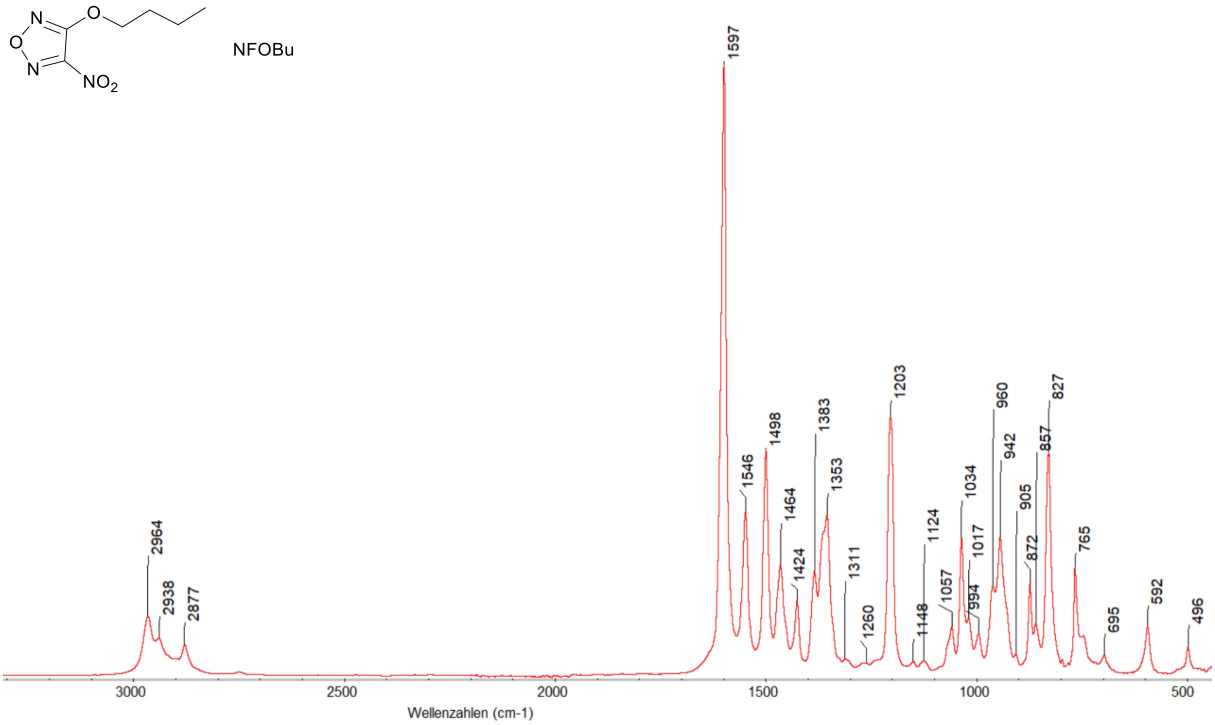
NFOBu
NO2
1597
1546
1498
1464
1424
1383
1353
1311
1260
1203
1148
1124
1057
1034
1017
994
960
942
905
872
857
827
765
695
592
496
2964
2938
2877
3000
2500
2000
1500
1000
500
Wellenzahlen (cm-1)

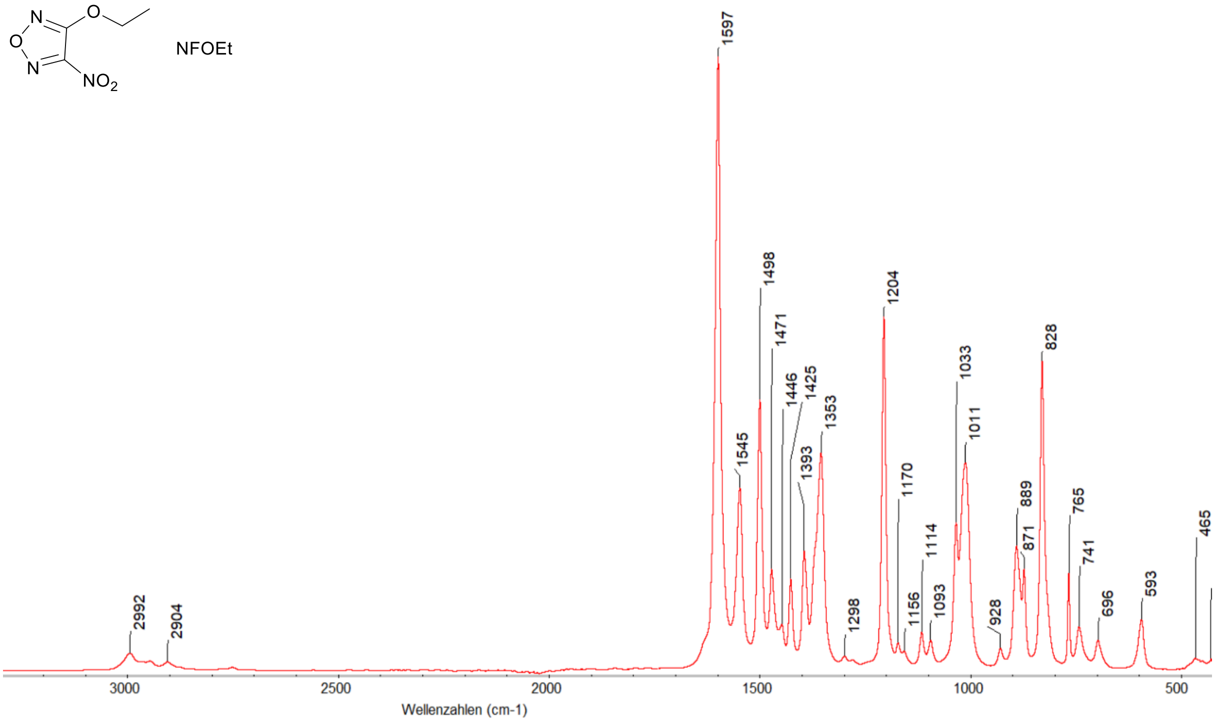
NFOEt
NO2
1597
1545
1498
1471
1446
1425
1393
1353
1298
1204
1170
1156
1114
1093
1033
1011
928
889
871
828
765
741
696
593
465
2992
2904
3000
2500
2000
1500
1000
500
Wellenzahlen (cm-1)

Mass spectra (S3)

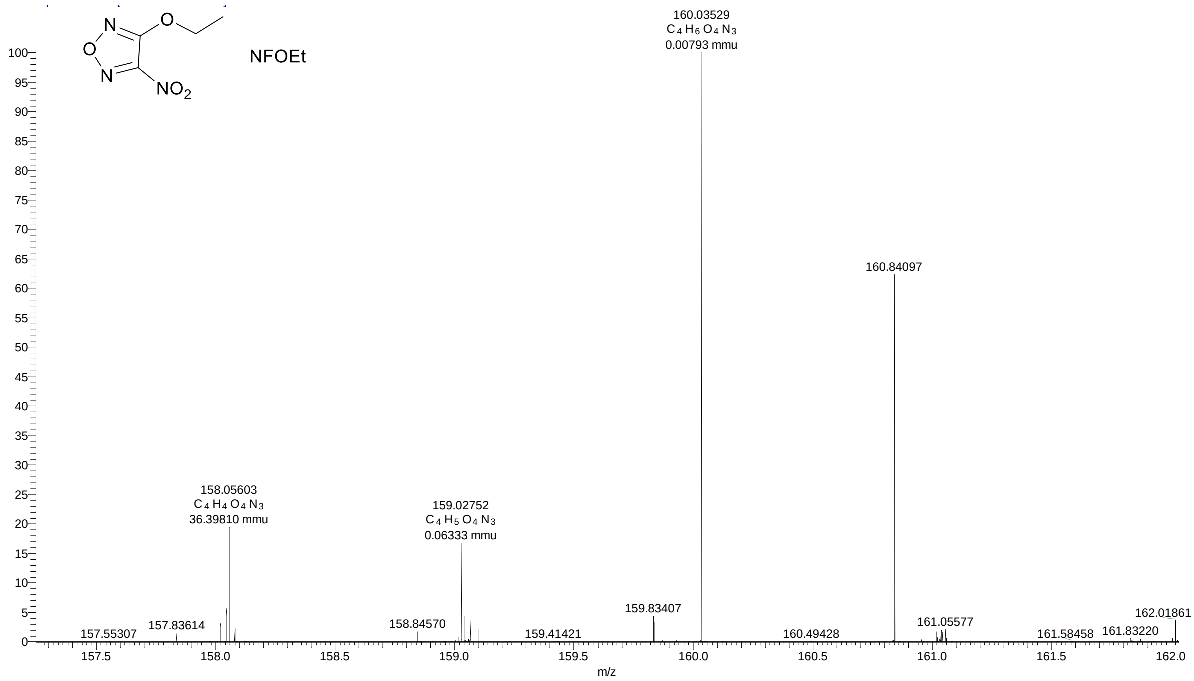

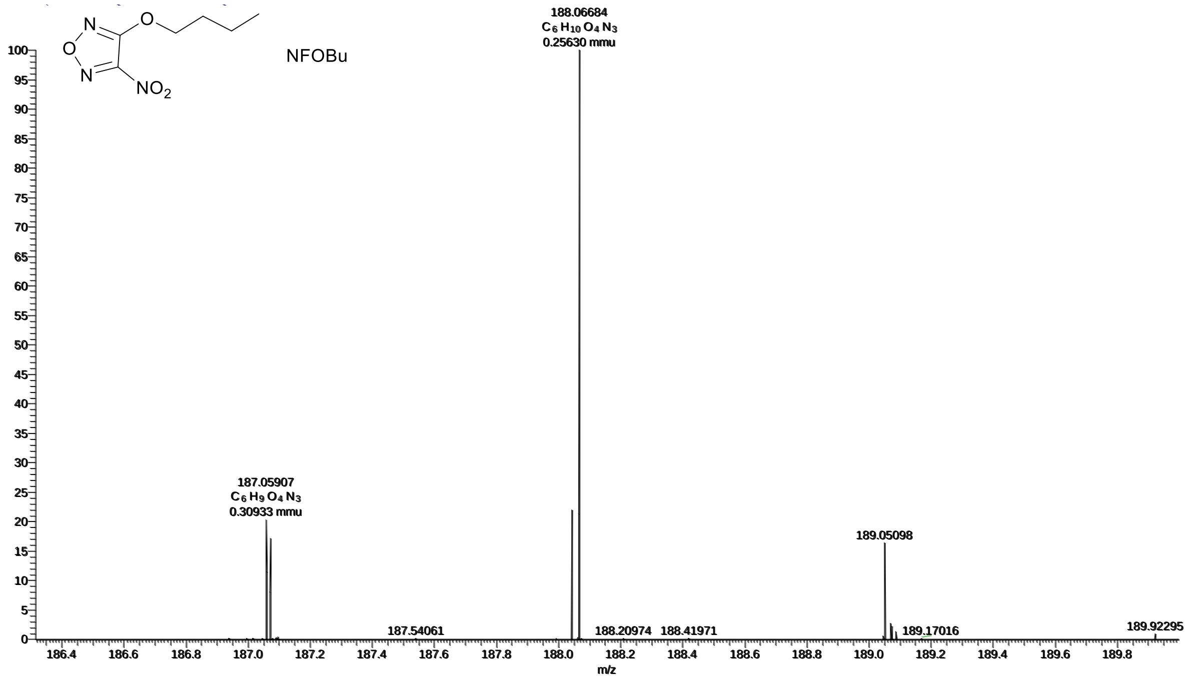

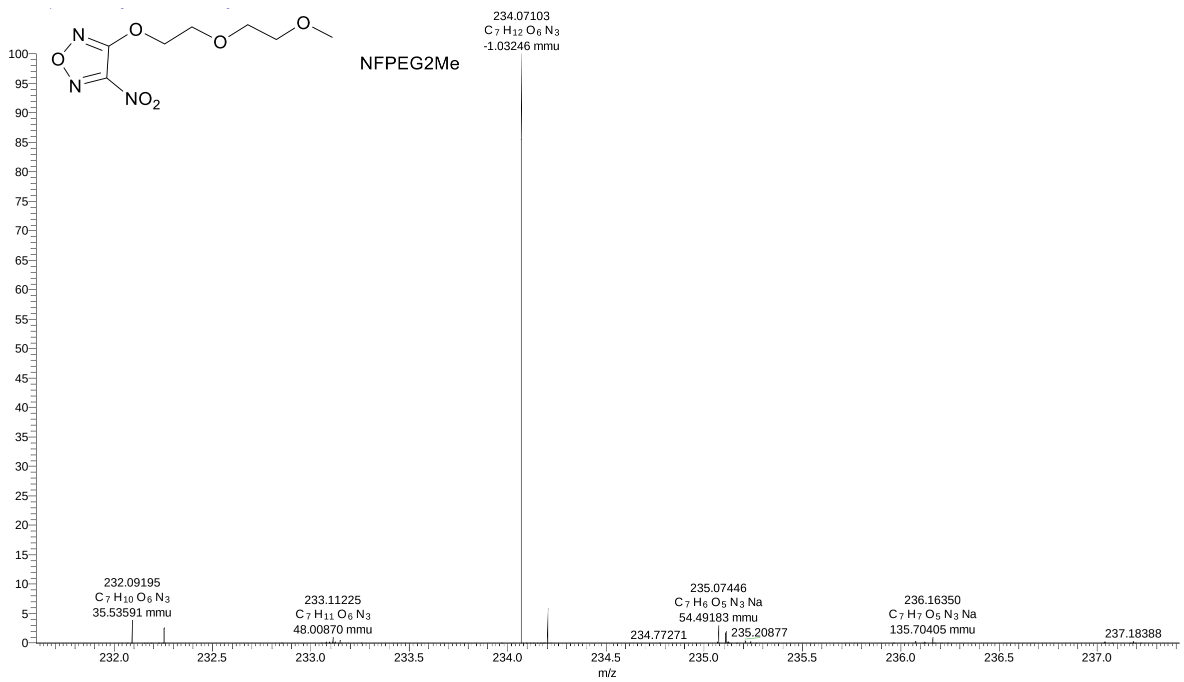
NFPEG2Me
234.07103
C 7 H 12 O 6 N 3
-1.03246 mmu
232.09195
C 7 H 10 O 6 N 3
35.53591 mmu
233.11225
C 7 H 11 O 6 N 3
48.00870 mmu
234.77271
235.07446
C 7 H 6 O 5 N 3 Na
54.49183 mmu
235.20877
236.16350
C 7 H 7 O 5 N 3 Na
135.70405 mmu
237.18388
m/z

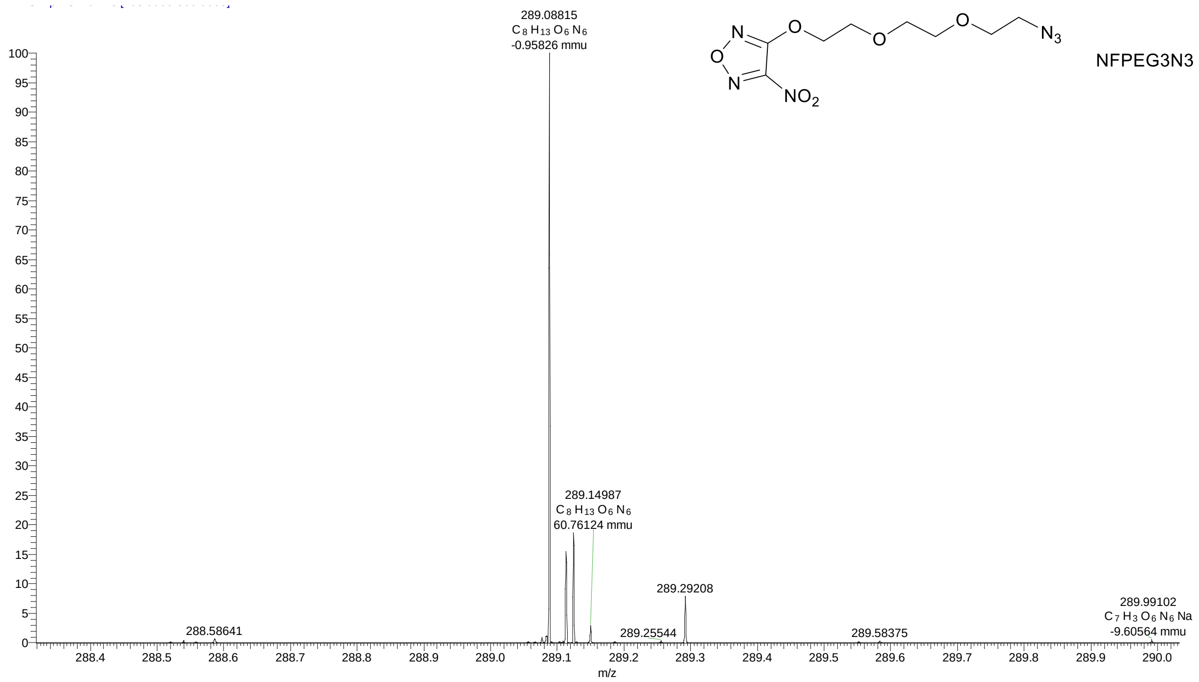
NFPEG3N3
289.08815
C 8 H 13 O 6 N 6
-0.95826 mmu
289.14987
C 8 H 13 O 6 N 6
60.76124 mmu
288.58641
289.25544
289.29208
289.58375
289.99102
C 7 H 3 O 6 N 6 Na
-9.60564 mmu
m/z

Pressure-tight crucible DSC (S4)

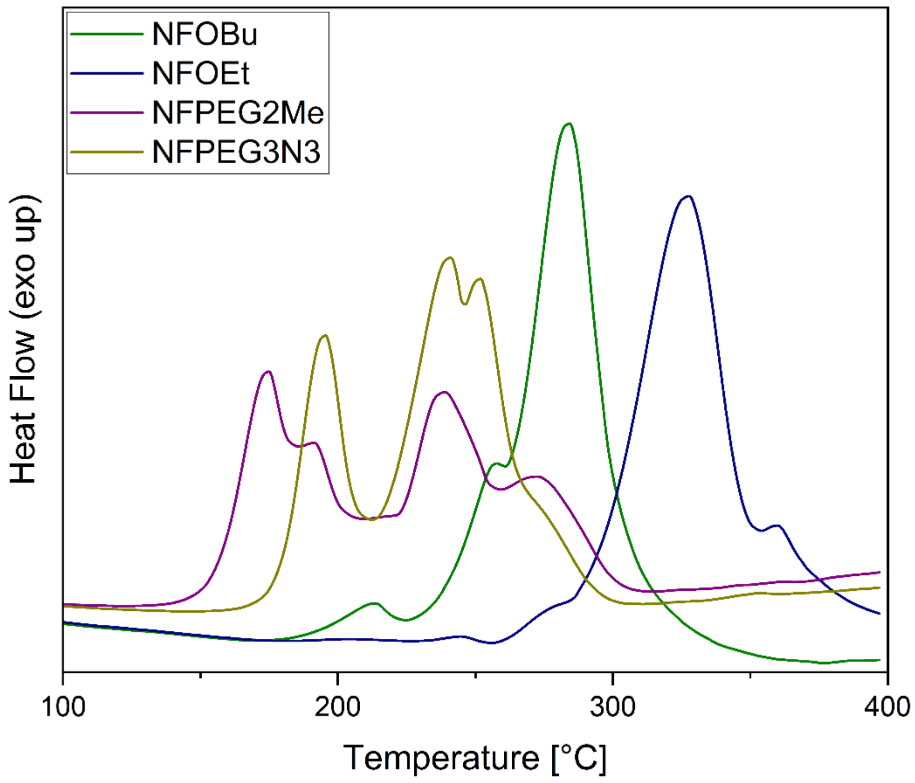

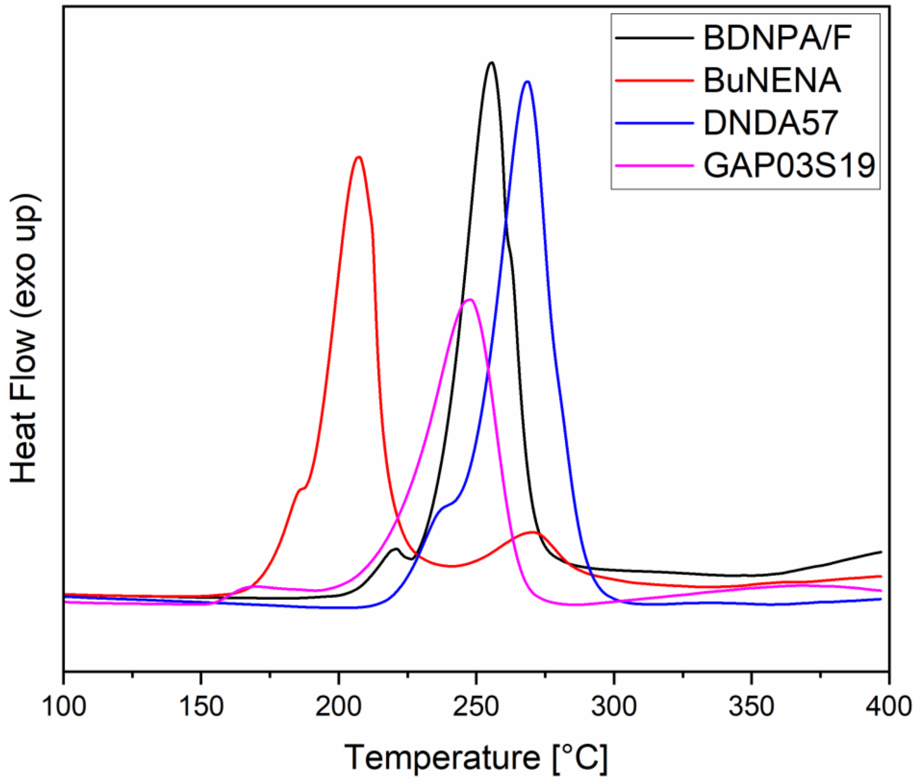

Perforated aluminum crucible DSC (S5)

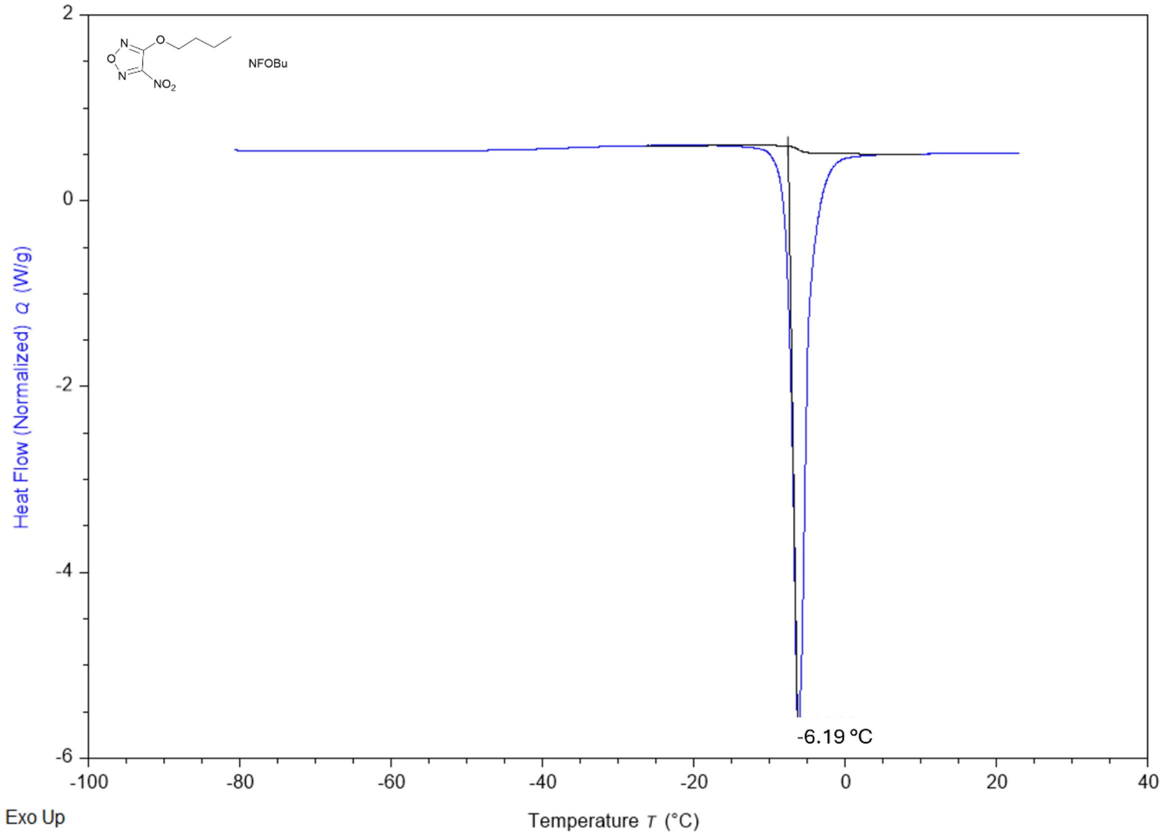

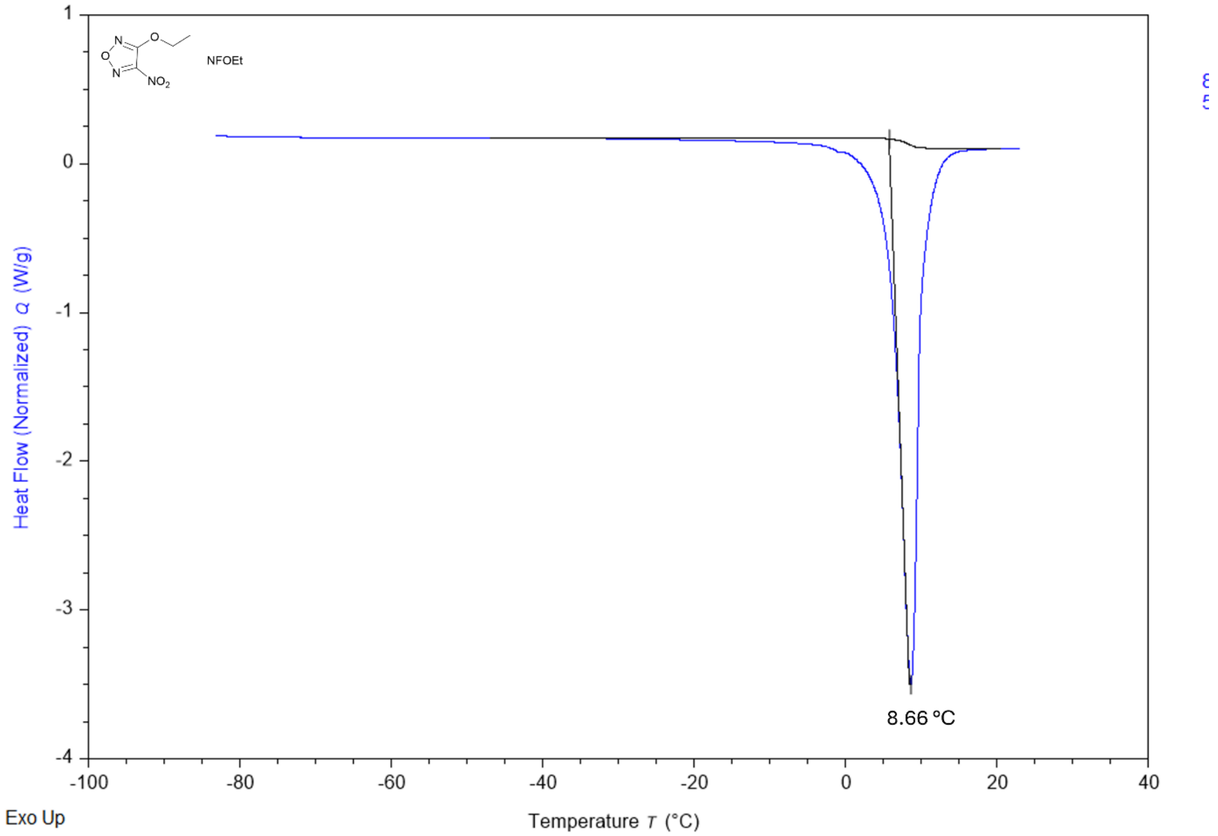

HPLC (S6)

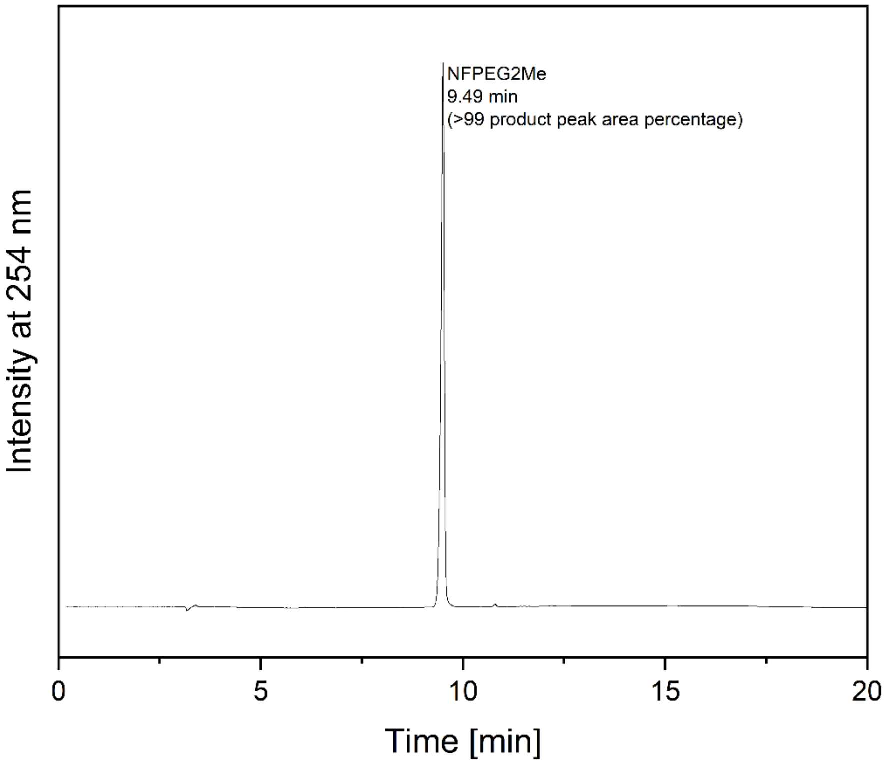

NFPEG2Me
9.49 min
(>99 product peak area percentage)
Intensity at 254 nm
0
5
10
15
20
Time [min]

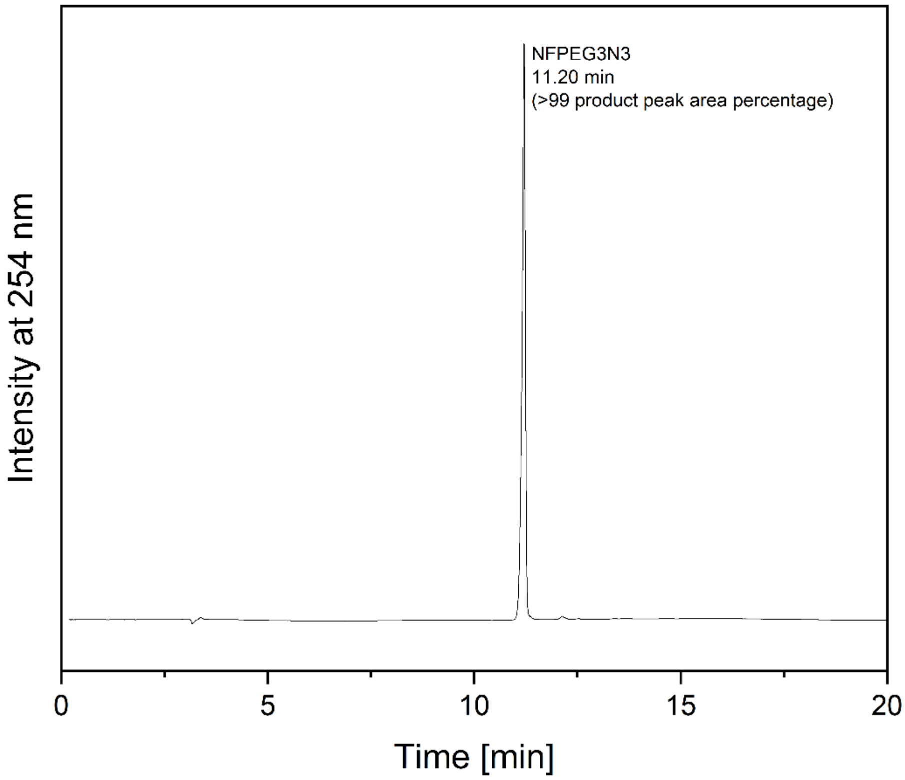

NFPEG3N3
11.20 min
(>99 product peak area percentage)
Intensity at 254 nm
0
5
10
15
20
Time [min]
